# Loss of schizophrenia-related miR-501-3p in mice impairs sociability and memory by enhancing mGluR5-mediated glutamatergic transmission

**DOI:** 10.1126/sciadv.abn7357

**Published:** 2022-08-19

**Authors:** Wenquan Liang, Yu Hou, Weiyuan Huang, Yunqian Wang, Tingyun Jiang, Xingbing Huang, Zhongju Wang, Fengchun Wu, Jiawei Zheng, Jie Zhang, Haiyan Ou, Shuyun Li, Junjiao Ping, Yuan Zhang, Junping Ye, Zhongwei Li, Qiong Yang, Jian Zhang, Xianzhen Zheng, Shufen Li, Xin-Hong Zhu, Rongqing Chen, Cunyou Zhao

**Affiliations:** ^1^ Department of Medical Genetics, School of Basic Medical Sciences, and Guangdong Technology and Engineering Research Center for Molecular Diagnostics of Human Genetic Diseases, Southern Medical University, Guangzhou, Guangdong, China.; ^2^ Senior Department of Pediatrics, The Seventh Medical Center of PLA General Hospital, Beijing, China.; ^3^ Department of Neurobiology, School of Basic Medical Sciences, Southern Medical University, China.; ^4^ The Third People’s Hospital of Zhongshan, Zhongshan, Guangdong, China.; ^5^ Department of Psychiatry, The Affiliated Brain Hospital of Guangzhou Medical University (Guangzhou Huiai Hospital), Guangzhou, China.; ^6^ The National Key Clinic Specialty, The Engineering Technology Research Center of Education Ministry of China, Guangdong Provincial Key Laboratory on Brain Function Repair and Regeneration, Department of Neurosurgery, Zhujiang Hospital, Southern Medical University, Guangzhou, China.; ^7^ Guangdong General Hospital, Guangdong Academy of Medical Science and Guangdong Mental Health Center, Guangzhou, China.; ^8^ Key Laboratory of Mental Health of the Ministry of Education, Guangdong-Hong Kong-Macao Greater Bay Area Center for Brain Science and Brain-Inspired Intelligence, and Guangdong Province Key Laboratory of Psychiatric Disorders, Southern Medical University, Guangzhou, China.; ^9^ Experimental Education/Administration Center, School of Basic Medical Science, Southern Medical University, Guangzhou, China.; ^10^ Department of Rehabilitation, Zhujiang Hospital, Southern Medical University, Guangzhou, China.

## Abstract

Schizophrenia is a polygenetic disease, the heterogeneity of which is likely complicated by epigenetic modifications yet to be elucidated. Here, we performed transcriptomic analysis of peripheral blood RNA from monozygotic twins discordant for schizophrenia and identified a schizophrenia-associated down-regulated microRNA, miR-501-3p. We showed that the loss of miR-501-3p in germline knockout (KO) male mice resulted in dendritic structure defects, glutamatergic transmission enhancement, and sociability, memory, and sensorimotor gating disruptions, which were attenuated when miR-501 expression was conditionally restored in the nervous system. Combining the results of proteomic analyses with the known genes linked to schizophrenia revealed that metabotropic glutamate receptor 5 (mGluR5) was one of the miR-501-3p targets and was elevated in vivo upon loss of miR-501. Treatment with the mGluR5 negative allosteric modulator 3-2((-methyl-4-thiazolyl) ethynyl) pyridine or the *N*-methyl-d-aspartate receptor antagonist 2-amino-5-phosphonopentanoic acid ameliorated the deficits observed in *Mir501*-KO mice. The epigenetic and pathophysiological mechanism that links miR-501-3p to the modulation of glutamatergic transmission provides etiological implications for schizophrenia.

## Teaser

Loss of miR-501-3p in mice induces sociability and memory impairments by enhancing excitatory glutamatergic transmission.

## INTRODUCTION

Schizophrenia is a complex neuropsychiatric disorder that is characterized by a complex range of symptoms and cognitive impairments and involves disturbances in neural circuity and synaptic plasticity. Although the estimated heritability of schizophrenia is high (60 to 80%), non-Mendelian features including a concordance rate in monozygotic (MZ) twins reaching 50% (*1*, *2*) indicate that the interplay between genetic and epigenetic or environmental factors plays an important role in mediating heterogeneity and disease susceptibility. Over the past decades, numerous schizophrenia-associated genetic variants have been identified by genome-wide association studies (GWASs) (*3*, *4*). These variants, each with a weak effect size and exhibiting combinatorial patterns of inheritance, probably convey risk for the disease through molecular networks and interactions. Of the major epigenetic factors, a class of small noncoding RNA molecules known as microRNAs (miRNAs), usually ~22 nucleotides in length, engages in posttranscriptional repression or mRNA destabilization of many target genes by base-pairing to the target mRNAs (*5*). Most miRNAs are evolutionarily conserved and are expressed in a developmental and tissue-specific manner. The functional importance of miRNA regulatory networks in neurodevelopment and brain physiology has been widely studied (*6*, *7*), and many studies have shown that these networks play essential roles in schizophrenia (*3*), providing for implication of miRNAs in the etiology and pathophysiology of schizophrenia. Many studies have reported that hundreds of miRNAs are dysregulated in postmortem brains or peripheral blood of people with schizophrenia (*7*–*18*); however, only a few miRNAs have been validated in rodent models, and many of their functions are still not known. Although miRNA profiles in postmortem brain remain to be determined, it is reasonable that change of miRNA expression extending beyond the brain could provide useful peripheral biomarkers for schizophrenia. Recent findings that substantial correlation between blood and brains in miRNA expression (*14*) supported feasibility to probe the miRNAs related to neural function in the peripheral blood transcriptome. Given that many miRNAs target synaptic proteins or signaling proteins regulating synapses (*6*, *7*, *18*–*20*), the implication of synaptic plasticity in cognition and the genetic association of miRNAs (such as *miR-137*) with schizophrenia raises the possibility that miRNAs contribute to the pathogenesis of schizophrenia through regulation of synaptic plasticity. However, this notion needs to be consolidated by more experiments testing the functions of risk miRNAs.

Accumulating evidences suggest that dysfunction of glutamatergic receptors is key to the etiology of schizophrenia (*21*). Glutamatergic receptors, including ionotropic and metabotropic receptors, are activated by excitatory glutamatergic neurotransmitter and elicit fast excitatory synaptic responses. Ionotropic glutamate receptors, including *N*-methyl-d-aspartate receptors (NMDARs), AMPA receptors (AMPARs), and kainite receptors, are ligand-gated cation channels. Metabotropic glutamate receptors (mGluRs) are G protein–coupled receptors composed of eight mGluR subtypes (mGluR1 to mGluR8) (*22*). mGluR5 can physically interact with NMDARs via scaffolding proteins (*21*). A substantial body of evidence indicates that mGluR5 is critically involved in learning and memory, which are also disrupted in schizophrenia (*23*, *24*). Moreover, the presence of genetic variants in the mGluR5 gene associated with schizophrenia (*25*, *26*) and up-regulated mGluR5 in the postmortem brain tissues of schizophrenia (*27*, *28*) indicate that alterations to mGluR5 signaling might contribute to cognitive dysfunction associated with schizophrenia. Several studies have demonstrated that miRNAs play regulatory roles in synaptic plasticity by directly targeting synaptic receptors or their downstream targets (*6*, *7*, *18*–*20*). For example, miR-501-3p has been reported to mediate the activity-dependent regulation of the AMPAR subunit GluA1 and long-lasting remodeling of rat dendritic spines (*20*). miR-501-3p was also reported to be linked to cognitive functioning in patients with Alzheimer’s disease (AD), where its serum expression level is significantly down-regulated compared to controls (*29*), suggesting that it contributes to synaptic plasticity related to cognitive functions, including learning and memory (*20*). The capacity that miRNAs regulate the expression of many genes simultaneously and influence synaptic functions at the pathway level makes miRNAs potentially very significant in the context of complex phenotype variations of neuropsychiatric disorders. However, because of the large numbers of miRNAs and their target genes, the regulatory molecular mechanism contributing to pathogenesis and development is largely unknown.

Examination of epigenetic profiles in MZ twins, particularly phenotype-discordant MZ twins, is an excellent strategy to understand the interplay between genetic and epigenetic or environmental factors in mediating individual differences in phenotype and disease susceptibility (*2*, *30*). MZ twins are exclusively matched for genetics, age, sex, cohort effects, maternal effects, and a common environment (*2*). In essence, the same sources of cells from MZ co-twins should exhibit identical genetic signatures, and epigenetic variations within MZ co-twin pairs indicate that epigenetic modifications may be particularly vulnerable to environmental influences, especially during embryonic development (*2*). In this study, we performed small RNA sequencing (sRNA-seq) of peripheral blood RNA obtained from schizophrenia-discordant (SDC) MZ twins and healthy concordant control (HCC) MZ twins to screen schizophrenia-associated differentially expressed miRNAs (DE-miRNAs). We then validated the function of miR-501-3p, a DE-miRNA, in a rodent mouse model and delineated the regulatory molecular mechanism by which the loss of schizophrenia-associated down-regulated miR-501-3p in male mice induced sociability, memory, and sensorimotor gating disruptions through mGluR5-mediated excitatory glutamatergic transmission enhancement. The epigenetic and pathophysiological mechanism that links miR-501-3p to the modulation of excitatory glutamatergic transmission provides etiological implications for schizophrenia.

## RESULTS

### Identification of schizophrenia-associated DE-miRNAs within SDC twins

**Fig. 1.**
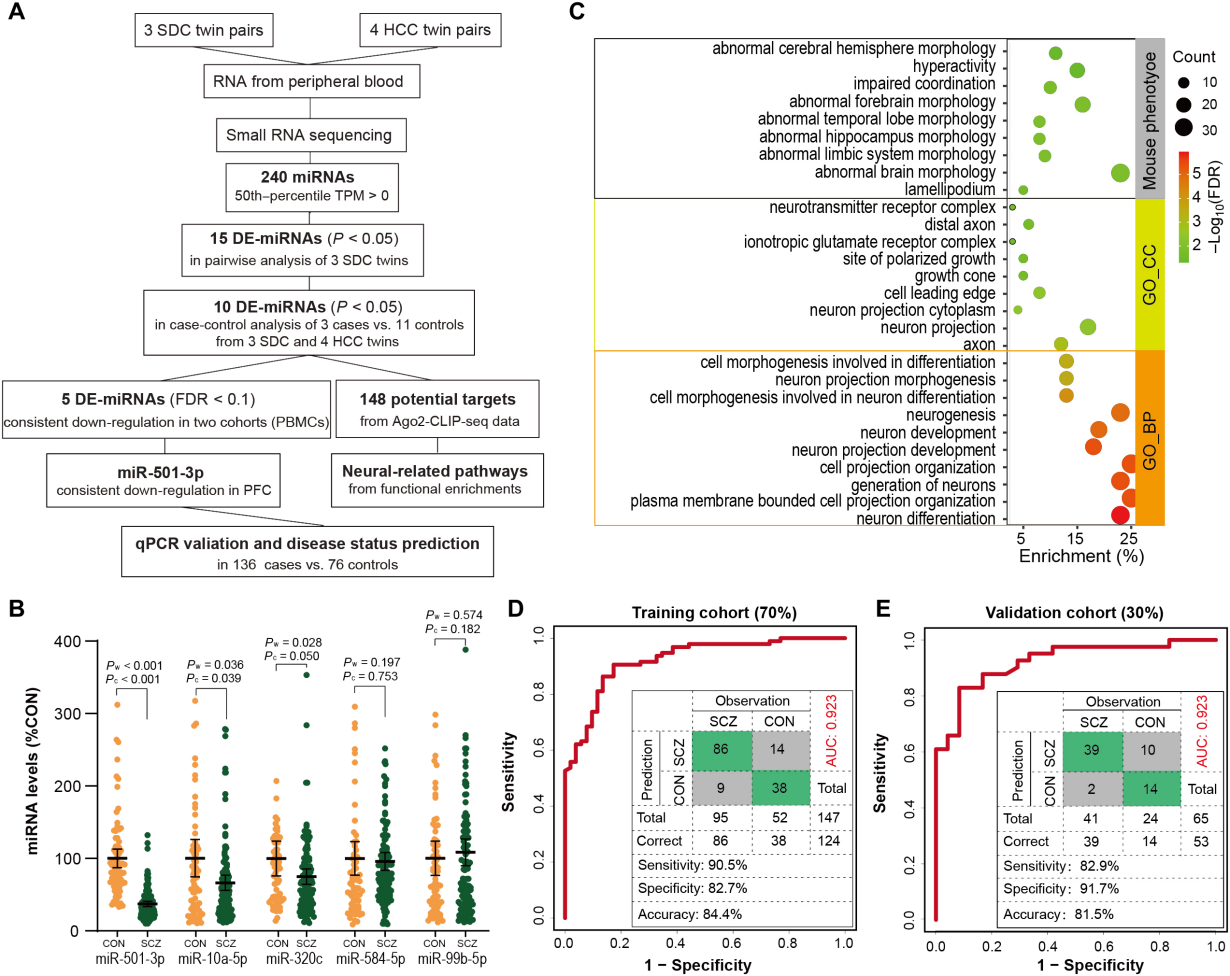
Screening of schizophrenia-associated miRNAs. (**A**) Discovery of DE-miRNAs in MZ twins. (**B**) The relative expression levels of five DE-miRNAs in peripheral blood samples from the validation cohort, namely, 136 patients with schizophrenia (SCZ) and 76 nonpsychiatric controls (CON). The expression levels in SCZ for miR-501-3p [CON mean = 100, 95% confidence interval (CI) = 87.08 to 112.9 and SCZ mean = 35.73, 95% CI = 32.08 to 39.37], miR-10a-5p (CON mean = 100, 95% CI = 74.32 to 125.68 and SCZ mean = 65.95, 95% CI = 55.42 to 76.48), miR-320c (CON mean = 100, 95% CI = 75.98 to 123.98 and SCZ mean = 74.83, 95% CI = 64.04 to 85.62), miR-584-5p (CON mean = 100, 95% CI = 75.92 to 123.04 and SCZ mean = 95.68, 95% CI = 83.34 to 108.01) and miR-99b-5p (CON mean = 100, 95% CI = 76.62 to 123.72 and SCZ mean = 108.45, 95% CI = 89.94 to 126.96) are shown as percentages relative to the mean level in the corresponding CON with 95% CI indicated by bars. Significant differences between two cohorts were determined with Mann-Whitney Wilcoxon test (*P*_w_) or with ANCOVA with age and sex as covariates (*P*_c_). (**C**) Functional enrichment analysis of 148 mRNA targets of 10 DE-miRNAs identified in Ago2-CLIP-seq. (**D** and **E**) Receiver-operating characteristic (ROC) curve analysis by binary logistic regression using transformed miR-501-3p level as an independent variable for distinguishing patients with SCZ from CON in training (D) and validation (E) cohort, respectively. ROC curve in logistic regression model including three variables of transformed miR-501-3p expression level, age, and sex was shown in fig. S1E.

To identify DE-miRNAs associated with schizophrenia, we performed sRNA-seq with a mean of 12 million 50–base pair (bp) single-end reads per sample from three pairs of SDC twins and four pairs of HCC twins (table S1). After quality control, mature miRNAs documented in miRBase release 20 (*31*) were identified and quantified using the miRDeep2 algorithm (fig. S1A) (*32*). Differential analysis of 240 unique miRNAs with a 50th percentile transcripts per million (TPM) > 0 among three MZ twin pairs identified 15 DE-miRNAs that displayed significant expression differences [|log_2_ fold change (log_2_FC)| > 0.585 and *P* < 0.05] in a pairwise analysis of three schizophrenia cases versus three healthy controls (three SDC twins for three versus three; Fig. 1A and table S2) using edgeR (*2*, *33*). We then performed case-control analysis of three schizophrenia cases (from three SDC twin pairs) versus 11 healthy controls by including four HCC twin pairs (3 versus 11) (*2*) and observed that 10 of 15 DE-miRNAs remained significantly associated with schizophrenia (|log_2_FC| > 0.585 and *P* < 0.05; table S2). Among them, five DE-miRNAs—miR-501-3p, miR-10a-5p, miR-320c, miR-584-5p, and miR-99b-5p—have been reported to display consistent schizophrenia-associated down-regulation [false discovery rate (FDR) < 0.1] in peripheral blood mononuclear cells (PBMCs) of an Australian sRNA-seq dataset containing 36 individuals with schizophrenia and 15 nonpsychiatric healthy controls (*12*) or of a miRNA microarray dataset containing 112 individuals with schizophrenia and 76 nonpsychiatric controls (*9*). We further used quantitative reverse transcription polymerase chain reaction (qRT-PCR) to validate the expression levels of the five DE-miRNAs in blood samples of an independent cohort containing 136 patients with schizophrenia and 76 nonpsychiatric controls and revealed that decreased miR-501-3p, miR-10a-5p, and miR-320c expression levels in schizophrenia remained significant on the basis of Mann-Whitney Wilcoxon test (*P*_w_ < 0.05) and analysis of covariance (ANCOVA) test with age and gender as covariates (*P*_c_ ≤ 0.05) (Fig. 1B).

The biological functions of miRNAs underlying the risk of schizophrenia could be mediated through posttranscriptional repression of gene expression via binding to the 3′ untranslated regions (3′UTRs) of target mRNAs. We then analyzed Argonaute 2-cross-linking immunoprecipitation (CLIP)-sequencing (Ago2-CLIP-seq) data (RNA-seq of miRNAs and mRNAs isolated from cross-linking immunoprecipitation with an antibody to Ago2) (*34*) to screen the potential targets of 10 DE-miRNAs. We identified 148 genes that were actively targeted by the above 10 DE-miRNAs through 3′UTR regions of the transcripts from Ago2-CLIP-seq analysis of developing human brains (*34*). These mRNA targets displayed significant associations with schizophrenia-associated DE genes (DEGs) reported in the PsychENCODE brain RNA-seq dataset [40 overlapping genes; *P* = 0.0123 and odds ratio (OR) = 1.6] (*28*) and schizophrenia risk genes (28 overlapping genes; *P* = 0.0020 and OR = 1.97; fig. S1B) (*3*, *4*, *35*–*37*). Moreover, functional enrichment analysis revealed that abnormal mouse phenotypes such as abnormal hippocampal morphology, hyperactivity, impaired coordination, and abnormal forebrain morphology; cellular components such as axon, neuron projection, neuron projection cytoplasm, and cell leading edge; and biological processes such as neuron differentiation, generation of neurons, neurogenesis, and neuron development were significantly enriched among these mRNA targets of DE-miRNAs (Fig. 1C and table S3), pointing to functional implications of DE-miRNAs in the pathogenesis of schizophrenia.

Of 10 DE-miRNAs identified, miR-501-3p was of particular interest for subsequent functional analyses because it was also reported to display consistent schizophrenia-associated down-regulation in an Australian PBMC sRNA-seq dataset (FDR = 0.0003) including 36 patients with schizophrenia and 15 nonpsychiatric controls (*12*) and in exosomes from postmortem prefrontal cortices (FDR = 0.025) of 7 individuals with schizophrenia and 13 nonpsychiatric controls (*13*, *16*). To evaluate the diagnostic potential of miR-501-3p, we trained a binary logistic regression model with 10-fold cross-validations [repeat 200 times: mean area under the receiver operating characteristic (ROC) curve (AUC) = 0.921 and mean accuracy = 0.847] on the training cohort (70%) and then applied the same statistical model to the validation cohort (30%) to further validate and confirm the diagnostic performance of miR-501-3p (Fig. 1, D and E, and fig. S1, C to E) using qPCR data from Fig. 1B. For the training cohort, the miR-501-3p signature achieved an AUC of 0.923, a sensitivity of 90.5%, and a specificity of 82.7% (Fig. 1D). Consistent with the training cohort, miR-501-3p achieved a robust performance in the validation cohort (AUC = 0.923, sensitivity = 82.9%, and specificity = 91.7%; Fig. 1E). Previously, miR-501-3p has been reported to be down-regulated in serum samples from patients with AD compared with healthy controls and reported to be positively correlated with disease progression (*29*). miR-501-3p is also expressed in the mouse brain (*38*, *39*). Together, these results suggest that miR-501-3p is a brain-relevant miRNA and may contribute to cognitive functions. However, the underlying gene regulatory networks of miR-501-3p in the context of schizophrenia remain unclear.

### Loss of miR-501 in male mice induced sociability, memory, and sensorimotor gating disruptions

**Fig. 2.**
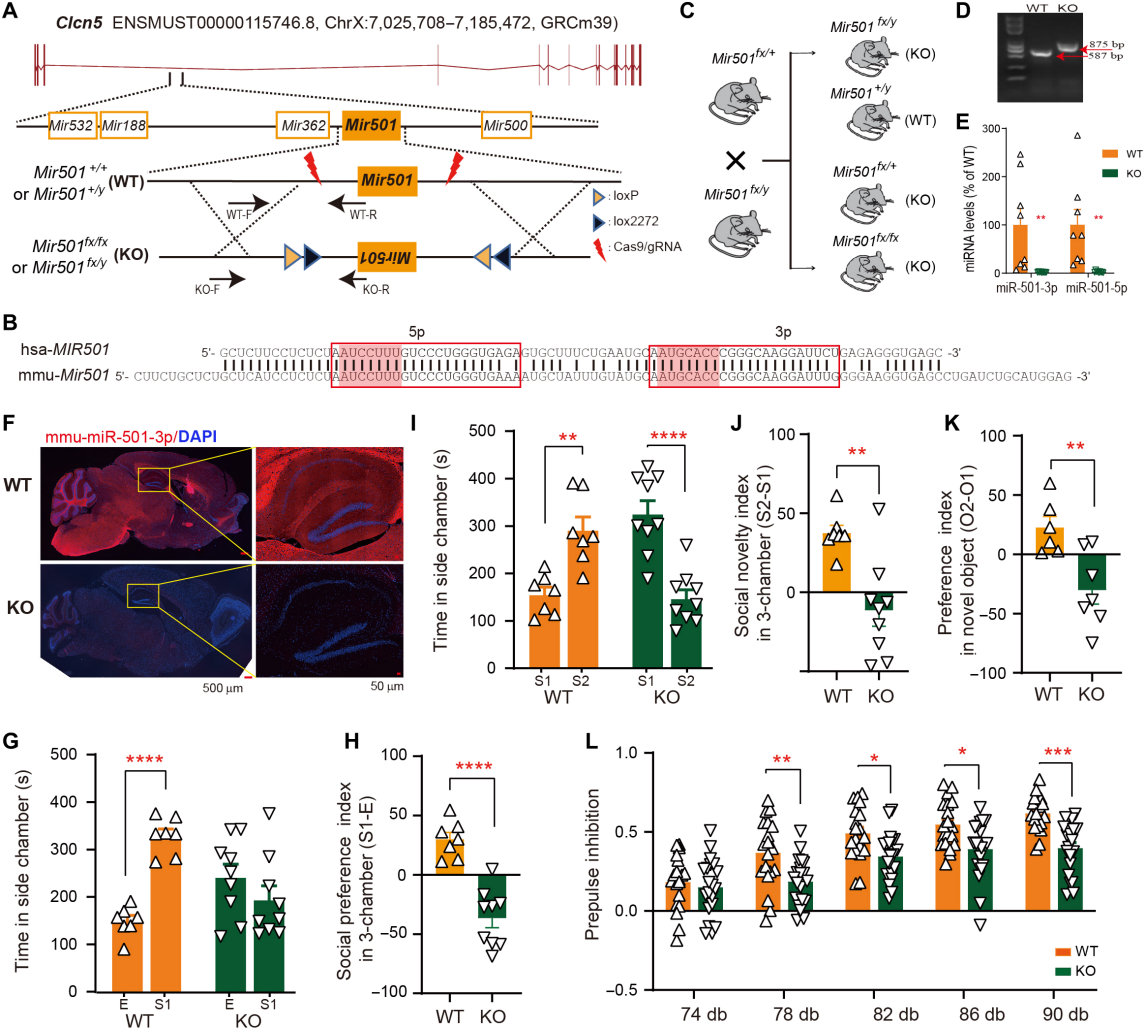
Loss of miR-501 in mice induced schizophrenia-like behavioral deficits. (**A**) Generation of the miR-501 conditional allele. The *Mir501* gene inverted in the targeting vector was flanked with the FLEx cassette containing one pair of loxP sites staggered with one pair of lox2722 sites. The arrows indicate the primer sets. (**B**) Sequence alignment of human and mouse mature miR-501 (red shading is used for the seeding region). (**C** to **E**) The generation of *Mir501*-KO mice obtained by crossing *Mir501^fx/+^* and *Mir501^fx/y^* mice (C) was examined by PCR analysis of DNA (D) and qRT-PCR analysis of RNA (two-tailed *t* test) (E). (**F**) Endogenous miR-501 was detected in brain from WT and KO mice by fluorescence in situ hybridization (FISH). The right image in each row shows the region outlined in the respective left merged image at high magnification in situ hybridization. Nuclei were counterstained with 4′,6-diamidino-2-phenylindole (DAPI). (**G** and **H**) Impaired social interaction in the three-chamber test was shown by the amount of time spent [two-way analysis of variance (ANOVA) with Bonferroni’s post hoc test] in a chamber with a novel mouse (S) versus an empty cage (E) (G) or by the social preference index (two-tailed *t* test) derived from the numerical difference between the time spent in the chamber with S1 and in the E divided by the total time spent × 100 (H). (**I** and **J**) Impaired social novelty recognition in the three-chamber test shown by the amount of time spent (two-way ANOVA with Bonferroni’s post hoc test) in the chamber with a new stranger (S2) versus an old stranger (S1) (I) or the social novelty index (two-tailed *t* test) (J). (**K**) Results of the novel object recognition test. O2 and O1, novel and familiar object. Two-tailed *t* test. (**L**) Impaired prepulse inhibition (PPI) in *Mir501* mice. Two-way ANOVA with Bonferroni’s post hoc test. All data represent the means ± SEMs from WT (orange) and KO (green) mice. *P < 0.05, **P < 0.01, ***P < 0.001, and ****P < 0.0001.

The human *MIR501* locus is located in the intronic region between exons 3 and 4 of the chloride channel 5 gene (*CLCN5*; ENST00000376088.3) on chromosome Xp11.23 (ChrX: 49,774,330 to 49,774,413; hg19; fig. S2). Mouse *Mir501*, also located in the intronic region between exons 3 and 4 of *Clcn*5 (ENSMUST00000115746.7) on ChrX: 7,159,469 to 7,319,233 (GRCm38; Fig. 2A), displays high similarity to human *MIR501* (Fig. 2B). To investigate the function of schizophrenia-related down-regulated miR-501-3p, we generated *Mir501*–knockout (KO) mice by inverting the *Mir501* gene and flanking it with the FLEx cassette (Fig. 2A), resulting in deficiencies in miR-501-3p and miR-501-5p expression in the absence of Cre. *Mir501*-KO (*Mir501^fx/y^*) mice along with their respective wild-type (WT; *Mir501^+/y^*) littermates were produced by breeding *Mir501 ^fx/+^* mice with *Mir501^fx/y^* mice (Fig. 2, C and D). We found significantly reduced levels of mature mmu-miR-501-3p and mmu-miR-501-5p in the brain upon inversion of *Mir501* (Fig. 2E), whereas the expression levels of *Clcn5* and four miRNAs (miR-532, miR-188, miR-362, and miR-500) whose genomic loci were near *Mir501* were not affected by *Mir501* inversion (fig. S2, B and C). Of five miRNAs located within the loci, only miR-501-3p was detected in our twin sRNA-seq data. miR-501-3p is the major mature form transcribed from the *MIR501* gene and is highly expressed in multiple tissue or specific neuronal types (fig. S2D) based on public RNA-seq datasets (*40*–*42*). We also detected that miR-501-3p was highly expressed in the cortex and hippocampal region of the mouse brain but that this expression was eliminated in *Mir501*-KO mice, as examined by in situ hybridization (Fig. 2F). Compared to WT littermates, *Mir501-*KO mice showed little difference in body or brain weight and showed no apparent morphological differences in major organ sections (heart, liver, lungs, and kidneys) according to hematoxylin and eosin staining (fig. S3). Considering that the *Mir501* locus is located on chromosome X, the following experiments were performed with male mice to avoid potential unexpected effects of random X-chromosome inactivation in female mice.

To determine whether *Mir501*-KO mice display abnormal behaviors, we first tested the mice in a three-chamber apparatus. WT mice (*Mir501^+/y^*) preferred to explore the first stranger mouse introduced [stranger 1 (S1)] over an empty cage more than the KO mice (*Mir501^fx/y^*) did, as measured by the time spent in each chamber (Fig. 2G) and the preference index derived from these parameters (Fig. 2H). When another stranger mouse (S2) was placed in the empty box, KO mice, unlike their WT littermates, preferred to explore S1 over S2 (Fig. 2, I and J). These results suggest that *Mir501*-KO mice are sociability impaired, displaying abnormal social preference and social novelty recognition. In the novel object recognition test, the KO mice preferred to spend less time at the novel object (O2) compared to the familiar object (O1) than their WT littermates (Fig. 2K), indicating the deficit in memory and cognition. Last, in the prepulse inhibition (PPI) paradigm, the KO mice and WT mice exhibited a similar acoustic startle response in 74 dB, but the KO mice exhibited a significantly lower PPI at 78, 82, 86, and 90 dB than their WT littermates (Fig. 2L). Together, these results from mice behavior tasks suggested that miR-501 deficiency induced sociability, memory, and sensorimotor gating disruptions.

### Loss of miR-501 resulted in synaptic deficits

**Fig. 3.**
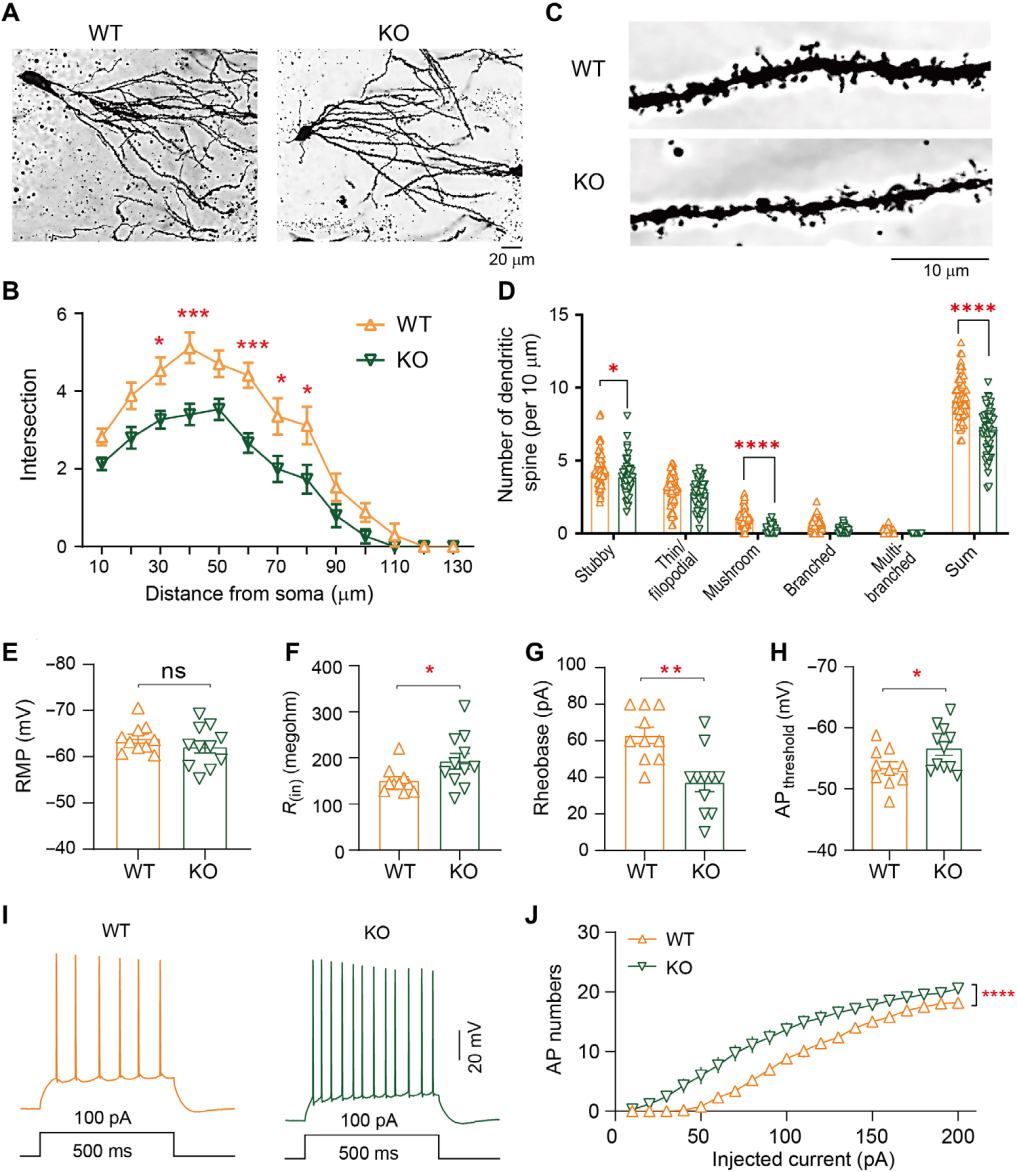
Loss of miR-501 in mice resulted in impaired dendritic growth and neuronal excitability. (**A** and **B**) Sholl analysis showing that, compared with neurons in WT mice, DG granule neurons in *Mir501*-KO mice exhibited decreased dendritic complexity. *n* = 17 slices from four WT mice and *n* = 15 slices from four KO mice; two-way ANOVA with Bonferroni’s post hoc test. (**C** and **D**) The spine densities were significantly decreased in *Mir501*-KO mice compared to WT littermate mice. *n* = 66 neurons and 48 neurons from four mice per group; two-way ANOVA with Bonferroni’s post hoc test. (**E**) Comparable resting membrane potentials (RMPs) of CA1 pyramidal neurons. *n* = 10 neurons from four WT mice and *n* = 11 neurons from four KO mice; two-tailed *t* test. (**F**) Enhanced input resistance [*R*_(in)_]. *n* = 10 neurons from four WT mice; *n* = 11 neurons from four KO mice; two-tailed *t* test. (**G**) Decreased rheobases. *n* = 10 neurons from four WT mice and *n* = 11 neurons from four KO mice; two-tailed *t* test. (**H**) Lower action potential (AP) thresholds. *n* = 10 neurons from four WT mice and *n* = 11 neurons from four KO mice; two-tailed *t* test. (**I**) Representative traces of AP firing evoked by injecting depolarizing currents. (**J**) Firing numbers plotted against increasing injected currents. Two-way ANOVA with Bonferroni’s post hoc test. All data represent the means ± SEMs from WT (orange) and KO (green) mice. *P < 0.05, **P < 0.01, ***P < 0.001, and ****P < 0.0001. ns, nonsignificant.

Given that miR-501-3p has been reported to modulate spine remodeling (*20*), the sociability and memory deficits observed in *Mir501*-KO mice may also involve defective synaptic structure or function. We thus performed Golgi staining using mouse hippocampal tissue at postnatal day 90 (P90) to determine whether the loss of miR-501 influenced dendritic structure or growth in vivo. Compared with the neurons in the hippocampal dentate gyrus (DG) region of WT littermates, the neurons from the KO mice exhibited reduced dendritic complexity and growth (Fig. 3, A and B). Moreover, the *Mir501*-KO mice showed reductions in the total spine density (stubby and mushroom-shaped spine types) of the hippocampal DG region relative to those in WT littermates (Fig. 3, C and D). These results together with miR-501-3p required for long-lasting spine remodeling (*20*), indicating that miR-501 plays essential roles in regulating synaptic structure or function.

**Fig. 4.**
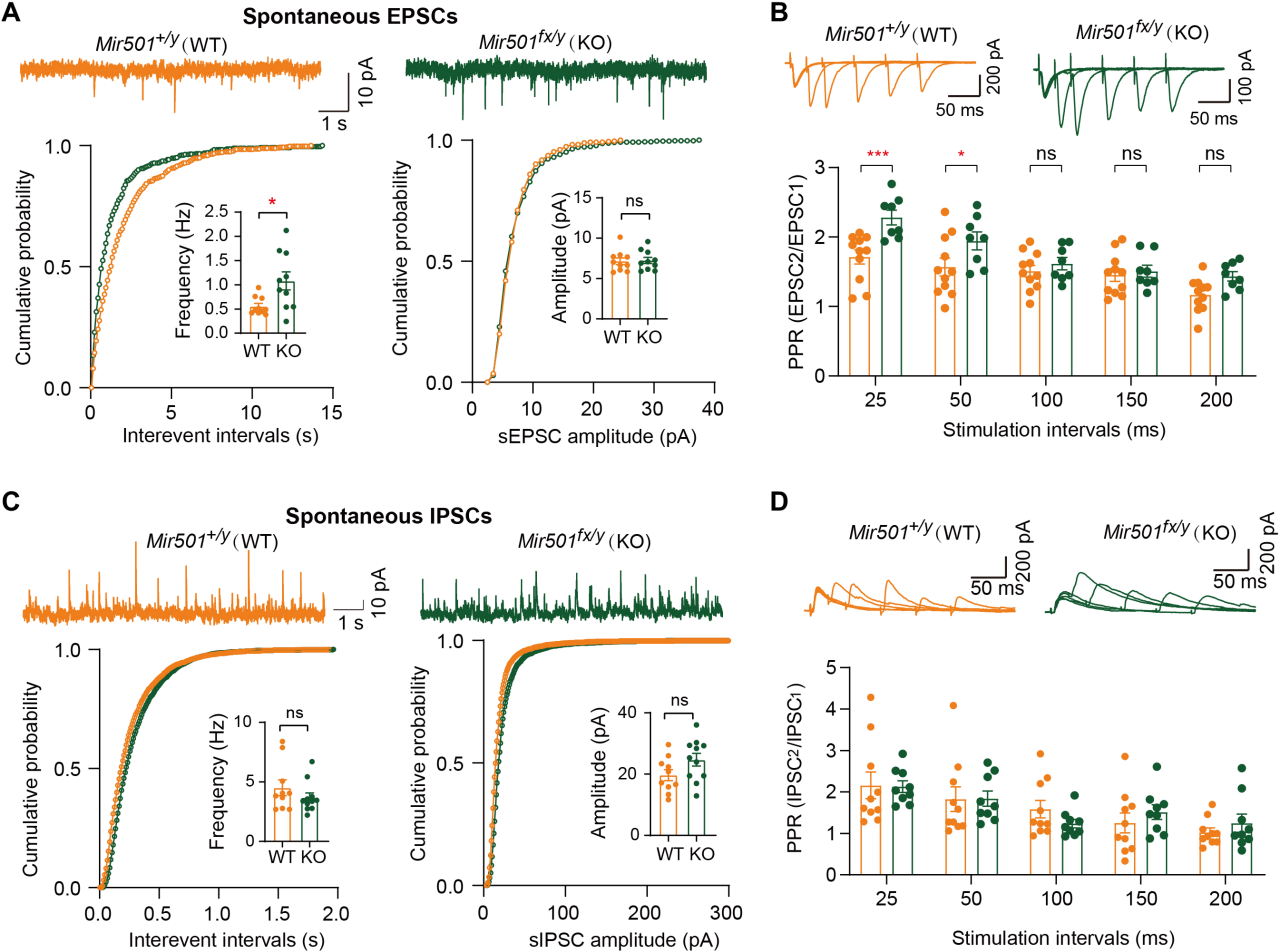
Loss of miR-501 in mice resulted in excitatory synaptic transmission impairment in CA1 hippocampal pyramidal neurons. (**A**) Representative recording traces and cumulative probability plots of sEPSCs showing that the frequencies (*P* = 0.0163 from two-tailed *t* test and *P* = 0.0181 from Kolmogorov-Smirnov test) but not amplitudes were enhanced in *Mir501*-KO hippocampal CA1 pyramidal neurons. *n* = 10 cells from four WT mice and *n* = 10 cells from four KO mice. (**B**) Representative traces of pair-pulse stimulation responses (top) and PPRs plotted against interstimulus intervals (bottom) (*P* < 0.0001 from two-way ANOVA with Bonferroni’s post hoc test). The PPR at the 25- or 50-ms interval was increased in *Mir501-*KO neurons*. n* = 8 cells from four KO mice and *n* = 11 cells from four WT mice. (**C** and **D**) No differences in sIPSC frequency (*P* = 0.2825 from two-tailed *t* test) or amplitude (*P* = 0.0886; *n* = 10 cells from WT mice and *n* = 11 cells from KO mice) (C) or in the PPR of evoked sIPSCs (*P* = 0.8628 from two-way ANOVA with Bonferroni’s post hoc test; *n* = 10 slices from WT mice and *n* = 9 slices from KO mice) (D) was observed between *Mir501*-KO and WT mouse neurons. All data represent the means ± SEMs from WT (orange) and KO (green) mice. *P < 0.05 and ***P < 0.001.

Next, we determined whether loss of miR-501 alters synaptic function. We observed that loss of miR-501 had little effect on the resting membrane potential (RMP) of hippocampal pyramidal neurons (Fig. 3E). However, increased input resistance (Fig. 3F) and decreased rheobase (Fig. 3G) were observed in KO mice compared with their WT littermates. The KO CA1 pyramidal neurons also showed a lower action potential (AP) threshold (Fig. 3H) but fired significantly more APs than WT neurons in response to depolarizing current injections (Fig. 3, I and J), indicating elevated excitability. We further observed that the spontaneous excitatory postsynaptic current (sEPSC) frequency, but not amplitude, of *Mir501*-KO pyramidal neurons was increased (Fig. 4A). The increased sEPSC frequency may also have been due to increased glutamate release probability, as pair-pulse ratios (PPRs) at 25- and 50-ms intervals were significantly enhanced for *Mir501*-KO neurons compared to WT neurons (Fig. 4B). To determine whether miR-501 deficiency alters γ-aminobutyric acid-ergic (GABAergic) transmission, we measured spontaneous inhibitory postsynaptic currents (sIPSCs) in pyramidal neurons. However, we did not observe any difference in sIPSC frequency or amplitude (Fig. 4C) or PPR amplitude (Fig. 4D) between the pyramidal neurons of *Mir501*-KO mice and their WT littermates. In summary, these results indicate that miR-501 plays vital roles in maintaining proper excitatory glutamatergic transmission.

### Rescue of miR-501 expression ameliorated behavioral and synaptic deficits

**Fig. 5.**
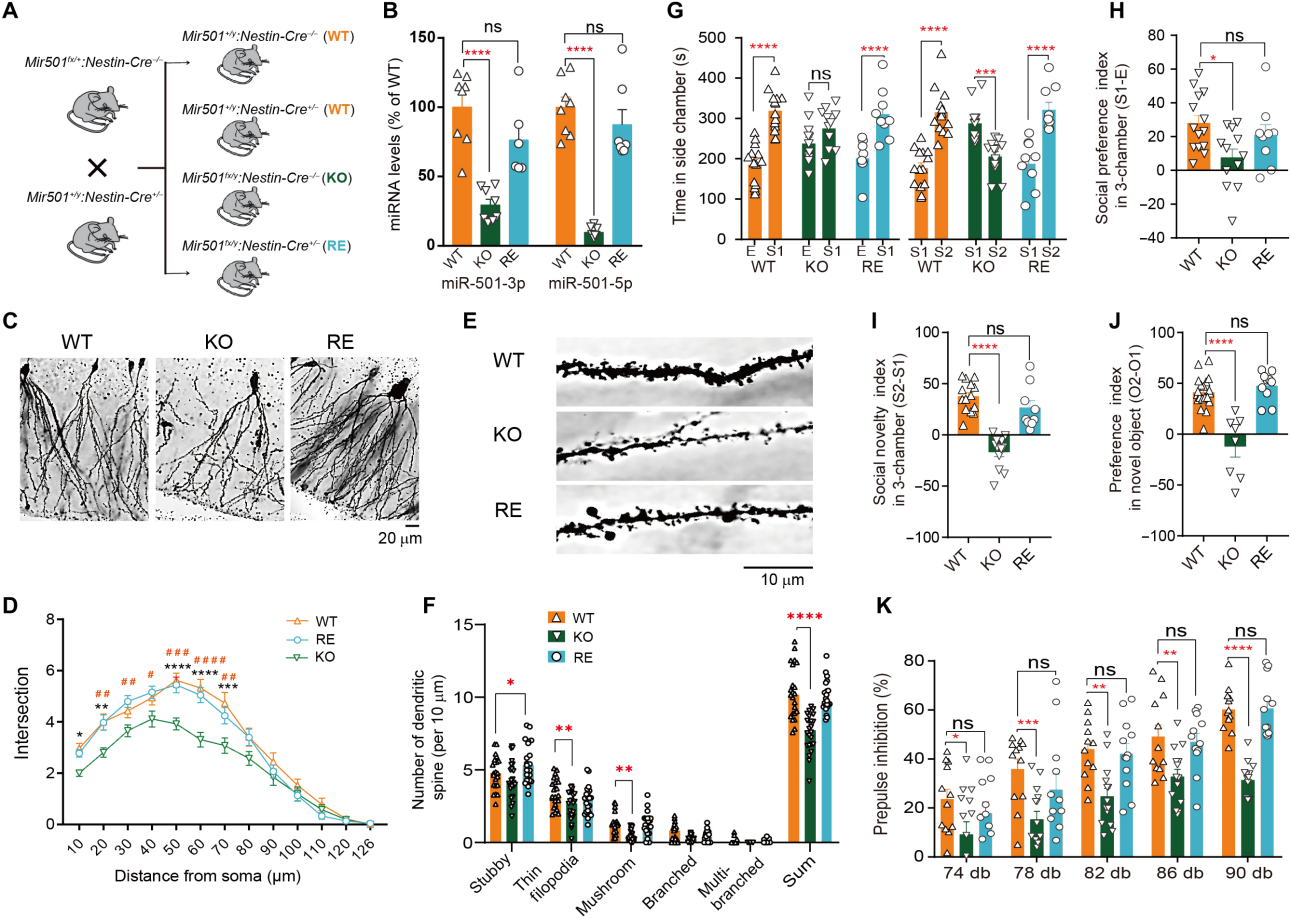
Rescue of miR-501 expression in mice ameliorated behavioral and synaptic deficits. (**A** and **B**) Schematic of the crosses used to generate conditional *Mir501*-RE mice. By crossing the mice with the *Nestin*-Cre mouse line, we specifically rescued *Mir501* in the central nervous system in *Mir501^fx/y^*:*Nestin*-*Cre^+/−^* mice (RE) (A) and validated the rescue by qRT-PCR of miR-501-3p and miR-501-5p expression (one-way ANOVA) in the hippocampus (B). (**C** and **D**) The reduced dendritic complexity of hippocampal DG granule neurons in *Mir501*-KO mice was rescued in *Mir501*-RE mice. *n* = 26 neurons from WT mice, *n* = 37 neurons from KO mice, and *n* = 45 neurons from RE mice. Significant differences were determined with two-way ANOVA with Bonferroni’s post hoc test shown with #*P* < 0.05, ##*P* < 0.01, ###*P* < 0.001, and ####*P* < 0.0001 for differences between KO and RE or *P < 0.05, **P < 0.01, ***P < 0.001, and ****P < 0.0001 for differences between KO and WT. (**E** and **F**) The reduced spine densities in *Mir501*-KO neurons were rescued in *Mir501*-RE neurons. *n* = 23 neurons from WT mice, *n* = 24 neurons from KO mice, and *n* = 23 neurons from RE mice. (**G**) Rescue of miR-501 in neurons of *Mir501*-RE mice attenuated the social preference deficits (left) and social novelty deficits (right) in *Mir501*-KO mice. (**H** to **J**) Rescue of miR-501 in neurons restored the social preference index (H), the social novelty index (I), and the novel object recognition index (J) in *Mir501-*KO mice. One-way ANOVA with Bonferroni’s post hoc test. (**K**) Rescue of miR-501 restored the decreased PPI observed in *Mir501-*KO mice. All data represent the means ± SEMs from WT (orange), KO (green), or RE (blue) mice. Significance of differences among WT, KO, and RE mice by two-way ANOVA with Bonferroni’s post hoc test in (B) to (G) and (K) and by one-way ANOVA with Bonferroni’s post hoc test (H) to (J) are marked by *P < 0.05, **P < 0.01, ***P < 0.001, and ****P < 0.0001.

To restore miR-501 expression, we crossed *Mir501^fx/+^*:*Cre^−/−^* (*Mir501*-KO) mice with *Mir501^+/y^*:*Nestin*-*Cre^+/−^* (*Mir501*-WT) mice that expressed Cre recombinase in neurons, allowing restoration of miR-501 expression in neurons (Fig. 5A). The restored miR-501 expression levels in *Mir501*-rescue (*Mir501^fx/y^*:*Nestin*-*Cre^+/−^*; *Mir501*-RE) mice, as well as in their KO (*Mir501^fx/y^*:*Nestin*-*Cre^−/−^*) and WT (*Mir501^+/y^*:*Nestin*-*Cre^−/−^* or *Mir501^+/y^*:*Nestin-Cre^+/−^*) littermates, were determined by qPCR (Fig. 5B). We then observed that the decreased dendritic complexity and spine density in *Mir501*-KO mice was ameliorated when the expression of miR-501 was rescued in *Mir501*-RE hippocampal DG neurons (Fig. 5, C to F). We further examined whether the rescue of miR-501 improved the abnormal behaviors observed in *Mir501*-KO mice. In the three-chamber test, the time spent exploring the empty cage, strange mice, or familiar mice was similar between *Mir501*-RE mice and their WT littermates (Fig. 5, G to I), indicating that rescue of miR-501 improved the impaired sociability behaviors in *Mir501*-KO mice. Furthermore, rescue of miR-501 restored the reduced novel object recognition index in KO mice (Fig. 5J); the RE mice exhibited a preference to spend more time at the novel object (O2) than the familiar one (O1) that was relatively similar to the preference of their WT littermates but stronger than that of their KO littermates. Last, *Mir501*-RE mice displayed a similar sensorimotor gating process as indexed by the PPI of the acoustic startle response compared with WT littermates (Fig. 5K). In summary, these results together indicate that rescue of miR-501 expression in neurons improved impairments in sociability, memory, and sensorimotor gating associated with the loss of miR-501.

**Fig. 6.**
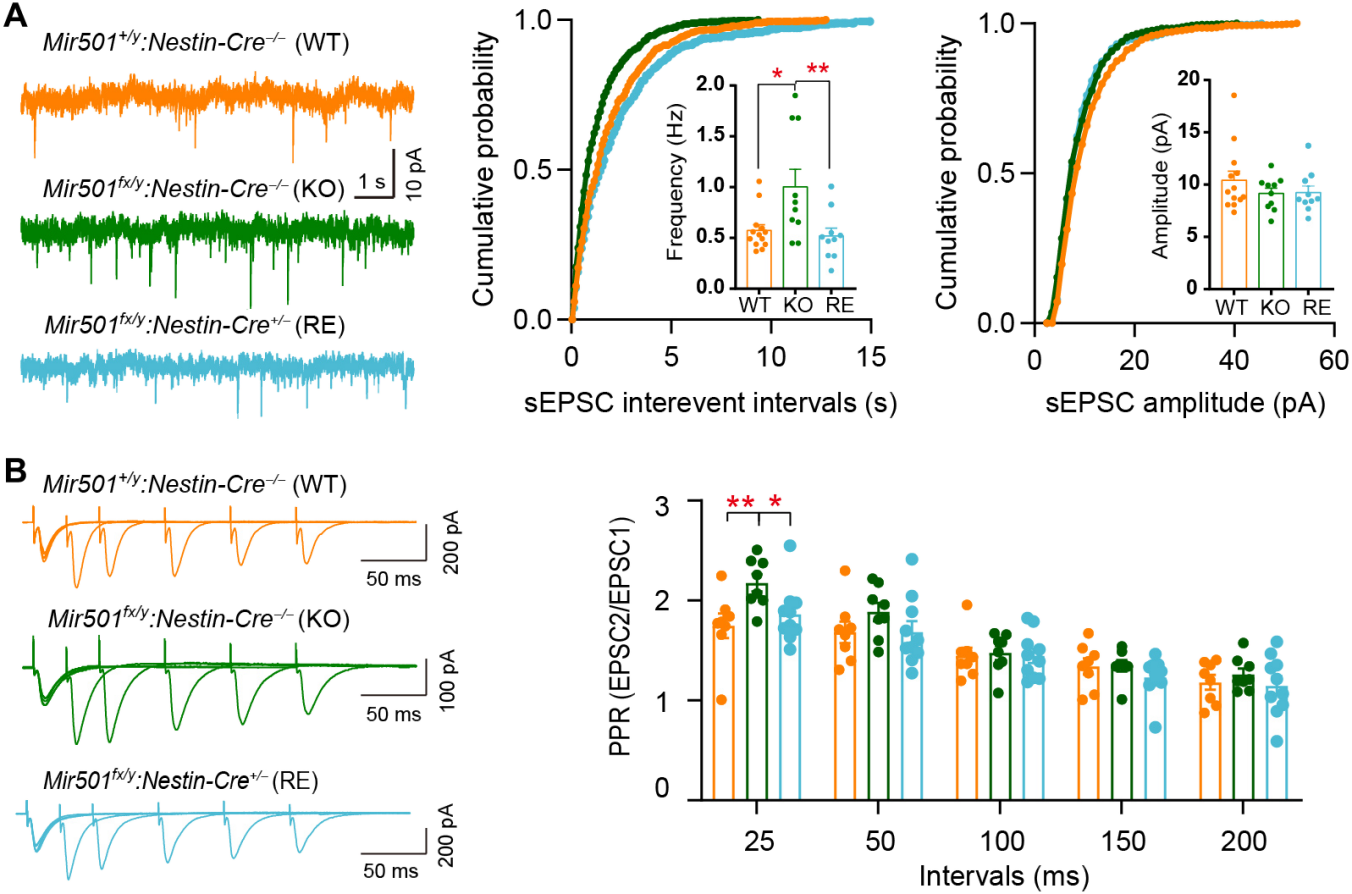
Rescue of miR-501-3p expression ameliorated the impaired sEPSCs in KO mice. (**A**) The increased sEPSC frequency in *Mir501*-KO neurons was reduced in *Mir501*-RE neurons and similar to that in WT neurons. *n* = 13 cells from WT mice, *n* = 10 cells from KO mice, and *n* = 10 cells from RE mice. *P* = 0.0067 for sEPSC frequency by one-way ANOVA with Bonferroni’s post hoc test and *P* = 0.0385 by Kolmogorov-Smirnov test. (**B**) Increased PPR at 25 ms in *Mir501-*KO neurons was restored when miR-501 expression was rescued in *Mir501*-RE mice. *P* = 0.0086 by two-way ANOVA with Bonferroni post hoc test. *n* = 8 neurons from WT mice, *n* = 8 neurons from KO mice, and *n* = 10 neurons from RE mice. All data represent the means ± SEMs. *P < 0.05 and **P < 0.01.

We further examined whether dysregulated synaptic function in *Mir501-*KO mice could be ameliorated in *Mir501*-RE mice. We observed that *Mir501*-RE mice had similar frequencies and amplitudes of sEPSCs as WT mice, both of which displayed lower frequencies of sEPSCs than *Mir501*-KO mice (Fig. 6A). Moreover, *Mir501-*RE mice exhibited restoration of the impaired PPR of the amplitudes of EPSCs at 25-ms interval observed in the KO mice (Fig. 6B), indicating that the presynaptic issue with glutamate release was improved when miR-501 expression was restored in *Mir501*-RE mice. Together, these results suggest that miR-501 plays essential roles in regulating excitatory synaptic potential and glutamatergic transmission.

### Genome-wide identification of mGluR5 as one of the in vivo targets of miR-501-3p

**Fig. 7.**
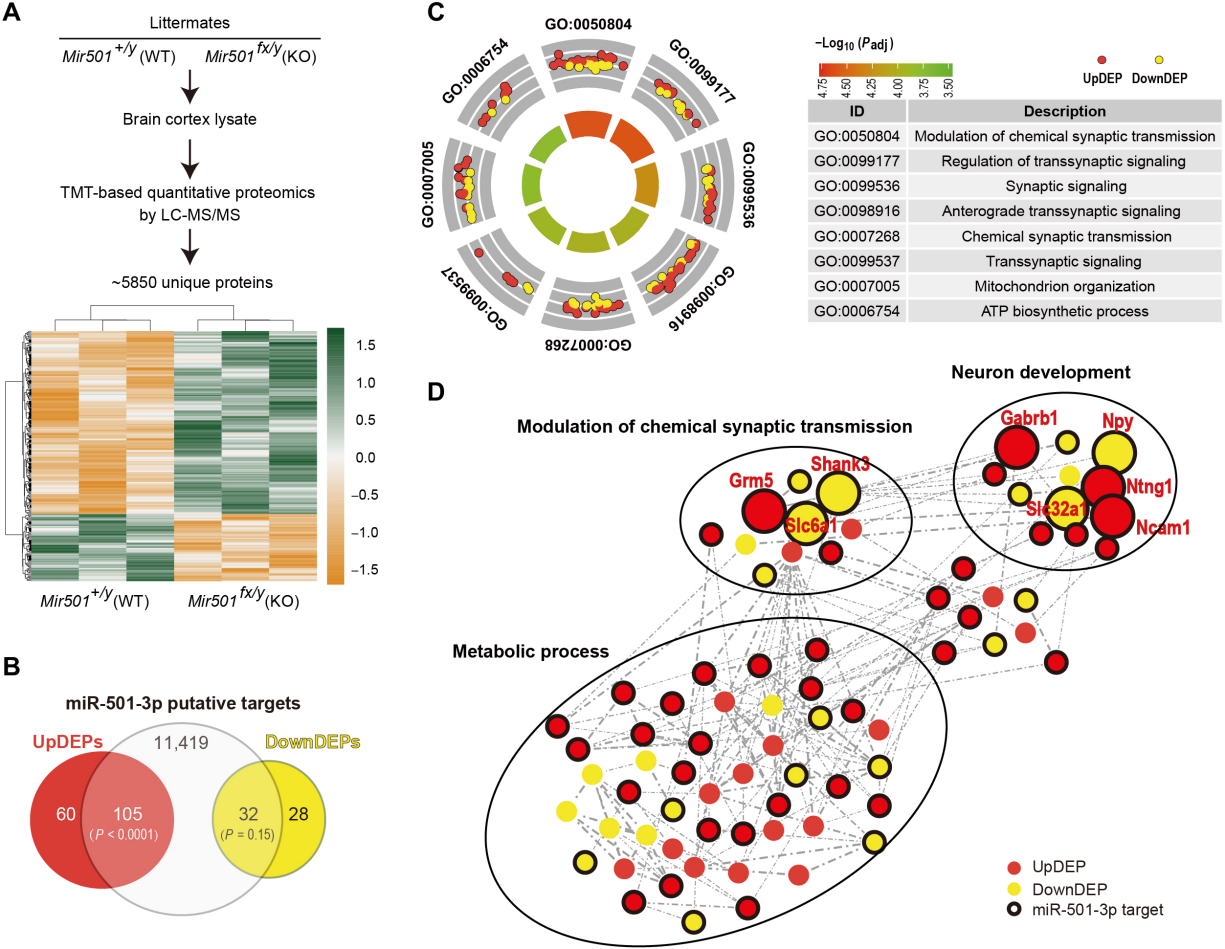
Genome-wide identification of in vivo protein targets of miR-501-3p. (**A**) Schematic workflow of quantitative proteomic analysis and heatmaps of 225 DE proteins in *Mir501*-KO brain samples compared with WT brains. Proteomic data were obtained from pooled cortex samples. *n* = 3 mice for each group (P90, male). LC-MS/MS, liquid chromatography–tandem mass spectrometry. (**B**) Overlap between putative miR-501-3p targets and DEPs. miR-501-3p putative targets (11,419 transcripts with conserved sites) significantly overlapped with up-regulated DEP (UpDEPs; 105 of 165 with *P* = 3.1 × 10^−6^ by hypergeometric test) but not with down-regulated DEP (DownDEPs; *P* = 0.15) identified in proteomic analysis. (**C**) GO-biological process enrichment analysis of 225 DEPs identified in the proteomic analysis. (**D**) Interactome of DEPs associated with loss of miR-501 in mice. The predicted protein-protein interaction network, generated with representative up-regulated DEPs (red dots) and down-regulated DEPs (yellow dots) that are miR-501-3p putative targets labeled with black circles, indicates the impact of miR-501-3p loss of function in modulation of chemical synaptic transmission, neuron development, and metabolic processes.

To investigate the mechanisms of miR-501 deficiency, we identified its targeted proteins whose levels were altered in *Mir501*-KO mice by tandem mass tagging (TMT)–based quantitative proteomics analysis (Fig. 7A). When comparing KO versus WT mice, we identified 225 DE proteins (DEPs; |*z* score| > 1.5 and *P* < 0.05; table S4) encoding 165 up-regulated proteins and 60 down-regulated proteins in *Mir501^fx/y^* cortexes (Fig. 7B). Gene ontology (GO) analyses showed that these DEPs were substantially involved in modulation of chemical synaptic transmission, regulation of transsynaptic signaling, synaptic signaling, adenosine triphosphate (ATP) biosynthetic processes, etc. (Fig. 7C and table S5). Interactome analysis using selected DEPs revealed a complex gene regulatory network, suggesting that these DEPs might be correlated with each other and might result in dysregulated expression upon loss of miR-501 (Fig. 7D). Notably, the up-regulated proteins Grm5 (glutamate metabotropic receptor 5; or mGluR5) and Gabrb1 (γ-aminobutyric acid type A receptor β1 subunit) and the down-regulated protein Shank3 (shank postsynaptic density protein) in *Mir501-*KO mouse brains were involved in the modulation of chemical synaptic transmission and neuron development, which may partially explain the altered sociability and memory behaviors or impaired synaptic plasticity in mice upon loss of miR-501. These proteins have also been reported to play key roles in neuropsychiatric disorders (*21*, *43*, *44*).

**Fig. 8.**
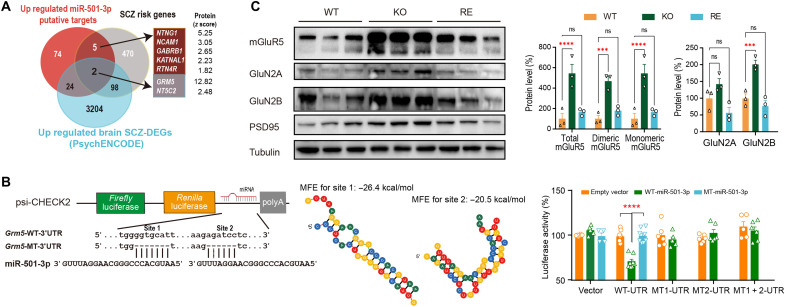
Screening and validation of mGluR5 as one of the direct targets of miR-501-3p. (**A**) Seven up-regulated targets were identified by overlapping the putative miR-501-3p targets up-regulated in *Mir501-*KO mice with the reported schizophrenia risk genes and schizophrenia-associated up-regulated genes identified in the PsychENCODE RNA-seq dataset. *z* score for proteins from TMT assay are shown. (**B**) Dual-luciferase reporter constructs with the psiCHECK-2 vector contain *Renilla* luciferase and *firefly* luciferase driven by two different promoters (left). miR-501-3p binding sites 1 and 2 in the *Grm5* 3′UTR (WT-UTR) were predicted by TargetScan and RNAHybrid and removed to generate the mutant *Grm5* 3′UTR (MT1-UTR, site 1 deleted; MT2-UTR, site 2 deleted; or MT1+2-UTR, sites 1 and 2 deleted). The minimum free energy (MFE) of the two binding sites was calculated by RNAHybrid (center). The effects of miR-501-3p on the *Grm5* 3′UTR were examined by cotransfection of a dual-luciferase reporter containing *Gmr5* WT or MT-3′UTR with the EGFPC1 empty vector (orange), miR-501-3p precursor (WT, green) or MT miR-501-3p (mutation of miR-501-3p seed region, blue) in the pEGFPC1 vector (right). ****P < 0.0001 by two-tailed *t* test. (**C**) Immunoblot analysis of mGluR5, GluNR2A, GluNR2B, PSD95, and tubulin in the synaptosome fraction of the hippocampi of *Mir501* WT, KO, and RE mice (left) and the relative intensity of each band compared to tubulin from WT, KO, or RE mice were normalized to the mean for WT mice (right). Significant differences among WT, KO, and RE mice are marked by ***P < 0.001, ****P < 0.0001, or ns from one-way ANOVA with Bonferroni’s post hoc test above the indicated comparison.

To identify the direct targets of miR-501, we overlapped the putative miR-501-3p targets (predicted by at least 1 of 10 parameters in miRWalk2.0; table S6) with these 225 DEPs and identified 105 up-regulated and 32 down-regulated miR-501-3p putative targets (table S4), and the up-regulated DEPs were significantly associated with miR-501-3p predicted targets (Fisher’s exact test, *P* < 0.001 and OR = 2.3; Fig. 7B). Among the 105 up-regulated miR-501-3p putative protein targets, only 1 target (*Cpne7*) exhibited significant up-regulation (FC > 1 and *P* < 0.05) at the mRNA level, as determined by RNA-seq (table S7), suggesting that miR-501 regulates its direct targets posttranscriptionally. We then explored whether miR-501-3p targets the reported schizophrenia candidate genes. Notably, we observed that both schizophrenia-associated up-regulated DEGs reported in the PsychENCODE brain RNA-seq dataset (26 overlapping genes; *P* < 0.001 and OR = 4.6) (*28*) and schizophrenia-risk genes (7 overlapping genes; *P* = 0.001 and OR = 6.9) (*35*, *36*) were significantly associated with miR-501-3p putative protein targets up-regulated in the *Mir501*-KO mouse brain (Fig. 8A). Of seven overlapping genes, *Grm5*, encoding mGluR5, was the most highly ranked *z* score of seven DEPs (*z* score = 12.8 and *P* = 0.008) and also displayed schizophrenia-associated up-regulation in the PsychENCODE brain RNA-seq dataset (log_2_FC = 0.043 and *P* = 0.02) (*28*). Moreover, mGluR5 was previously reported to be involved in the regulation of neural network activity and NMDAR function and plasticity (*21*, *45*–*49*). Therefore, *Grm5* was of interest for follow-up functional analyses.

To further confirm that miR-501-3p regulates the expression of mGluR5 through the predicted binding sites in the 3′UTR of *Grm5* (Fig. 8B), we cloned a mouse *Grm5-*WT 3′UTR and 3′UTRs with one or two deletions at the predicted miR-501-3p binding sites (MT1, MT2, and MT1+2) into the psiCHECK-2 dual-luciferase expression vector. We also cloned a WT-miR-501-3p precursor or MT-miR-501-3p precursor lacking the seeding region into the pEGFPC1 vector. We observed that the luciferase activity from the *Grm*5-WT 3′UTR was reduced in cells cotransfected with the WT-miR-501-3p precursor compared with the empty vector control (Fig. 8B, right), but it was rescued remarkably when the cells were cotransfected with the MT-miR-501-3p precursor. Moreover, no significant change was observed in the miR-501-3p binding site–deleted *Grm5*-MT 3′UTR reporter (MT1, MT2, or MT1+2) cotransfected with the WT-miR-501-3p precursor compared to the reporter cotransfected with the empty pEGFPC1 vector control.

We then observed that both dimeric and monomeric mGluR5 were up-regulated in the synaptosome fractions of the hippocampi and cortexes of *Mir501^fx/y^* (KO) mice compared with their WT littermates (fig. S4). When the expression of miR-501 was restored in neurons, the up-regulated mGluR5 expression in the synaptosome fractions of *Mir501*-KO mouse brains was diminished in *Mir501*-RE mice (Fig. 8C), pointing that *Grm5* is a specific target of miR-501-3p. In addition, we also observed that the NMDAR subunit GluN2A and GluN2B expression levels were up-regulated in *Mir501*-KO mouse brains, and these up-regulations were diminished when miR-501 expression was restored in *Mir501*-RE mice neurons (Fig. 8C), which may explain the hyperfunction of glutamatergic neuron in *Mir501*-KO mice.

### Inhibition of mGluR5 by MTEP diminished behavioral and synaptic deficits in *Mir501*-KO mice

**Fig. 9.**
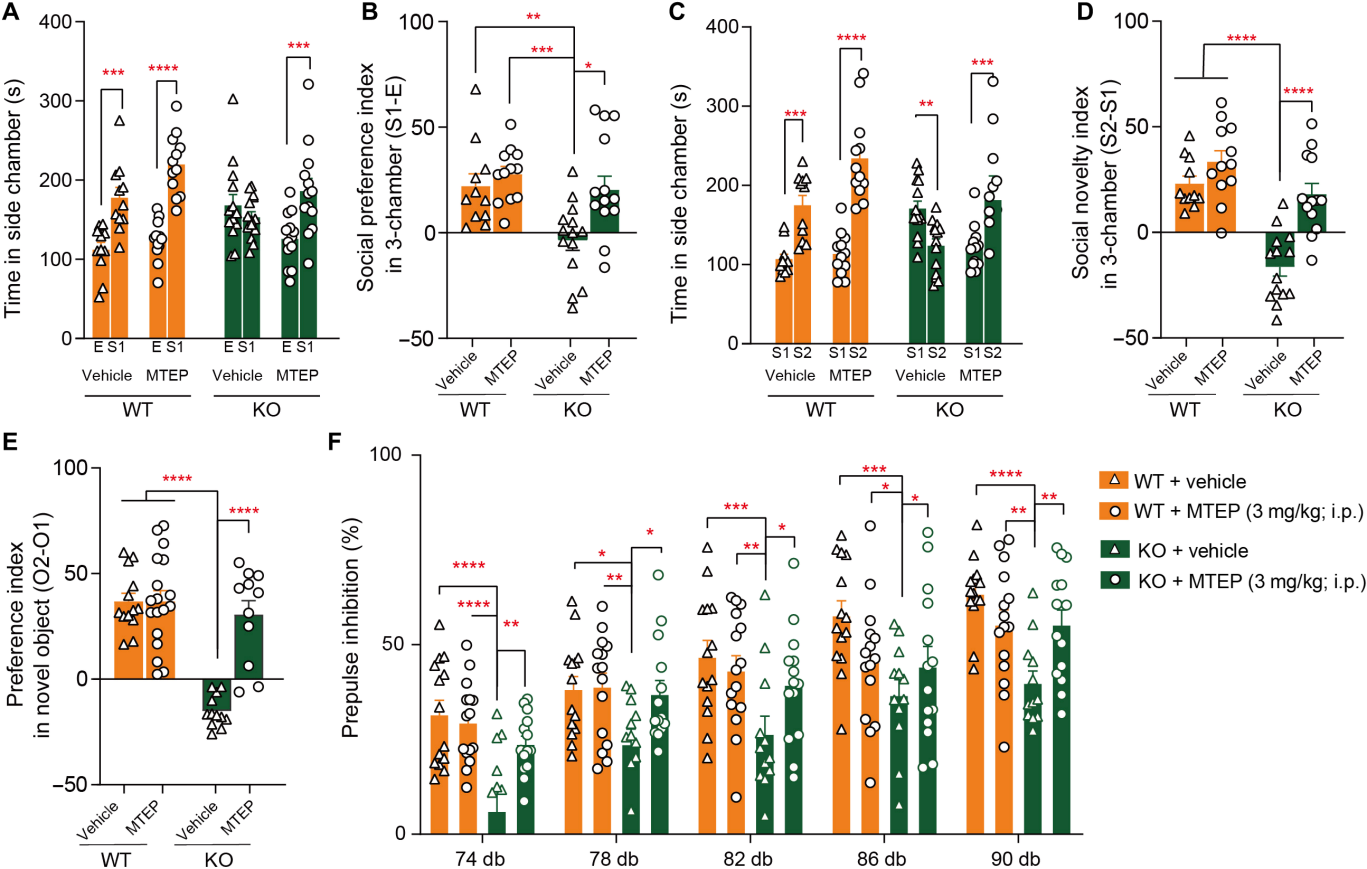
Inhibition of mGluR5 by MTEP diminished behavioral deficits associated with the loss of miR-501. (**A**) Social preference deficits in *Mir501*-KO mice were attenuated in the three-chamber test after treatment with MTEP. *P*_genotype_ = 0.992, *P*_drug_ = 0.165, and *P*_genotype/drug_ = 0.051 from three-way ANOVA with Bonferroni’s post hoc test. WT, *n* = 11 for vehicle and *n* = 12 for MTEP; KO, *n* = 14 for vehicle and *n* = 13 for MTEP. (**B**) The social preference index in *Mir501*-KO mice was restored after treatment with MTEP. *P*_genotype_ = 0.0029 and *P*_drug_ = 0.0175 from two-way ANOVA with Bonferroni’s post hoc test. (**C**) Social novelty deficits in *Mir501*-KO mice were attenuated in the three-chamber test after treatment with MTEP. *P*_genotype_ = 0.191 and *P*_drug_ = 0.033 from three-way ANOVA with Bonferroni’s post hoc test. (**D**) The social novelty index in *Mir501*-KO mice was restored after treatment with MTEP. *P*_genotype_ < 0.0001, *P*_drug_ < 0.0001, and *P*_interaction_ = 0.0324 from two-way ANOVA with Bonferroni’s post hoc test. (**E**) The preference index in *Mir501*-KO mice was restored in the novel object recognition test after treatment with MTEP. *P*_genotype_ < 0.0001, *P*_drug_ < 0.0001, and *P*_interaction_ < 0.0001 from two-way ANOVA with Bonferroni’s post hoc test. WT, *n* = 13 for vehicle and *n* = 18 for MTEP; KO, *n* = 12 for vehicle and *n* = 11 for MTEP. (**F**) The decreased PPI observed in *Mir501*-KO mice was restored after treatment with MTEP. *P*_genotype_ < 0.0001, *P*_drug_ = 0.019, and *P*_genotype/drug_ < 0.0001 from three-way ANOVA with Bonferroni’s post hoc test. WT, *n* = 14 for vehicle and *n* = 15 for MTEP; KO, *n* = 12 for vehicle and *n* = 14 for MTEP. All data represent the means ± SEMs. *P < 0.05, **P < 0.01, ***P < 0.001, and ****P < 0.0001.

**Fig. 10.**
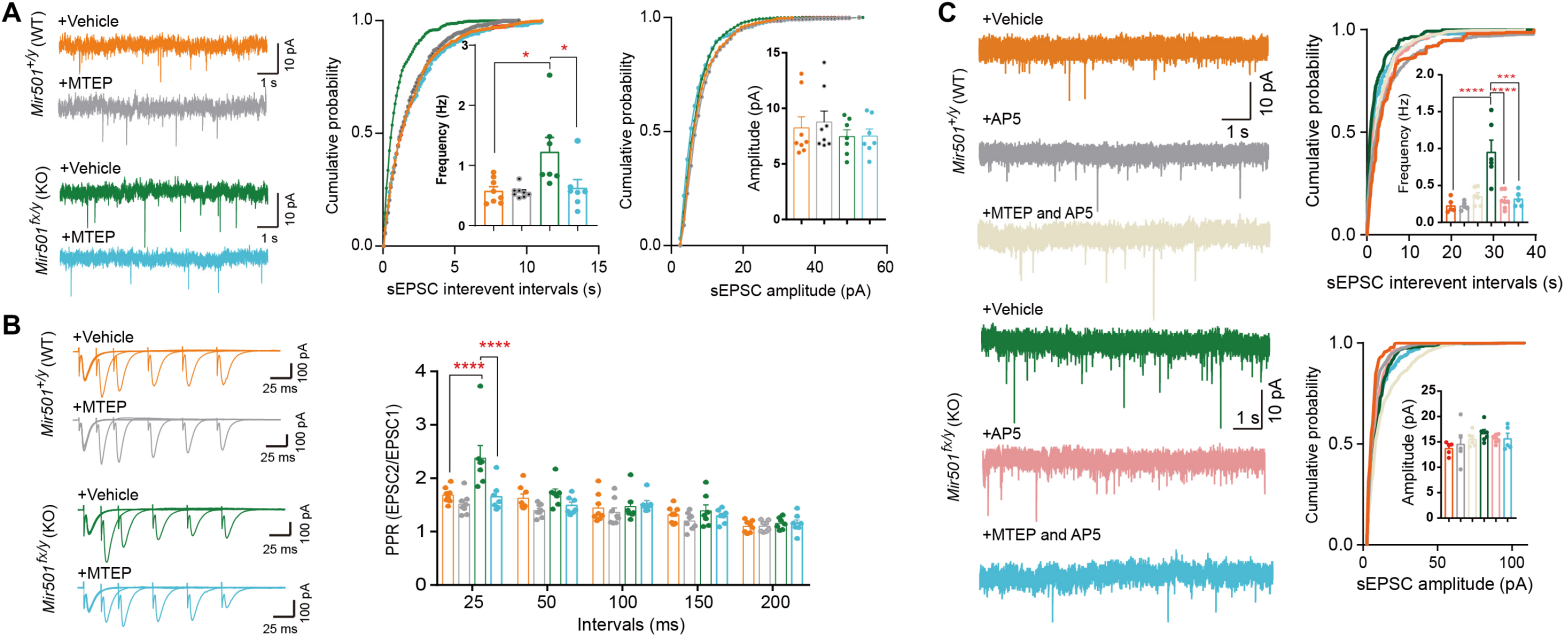
Effects of MTEP or/and AP5 on the impaired sEPSCs in *Mir501*-KO mice. (**A**) The sEPSC frequency of *Mir501*-KO neurons was reduced by treatment with MTEP and was similar to that of vehicle- or MTEP-treated WT neurons. *n* = 8 neurons from WT mice treated with vehicle or MTEP and *n* = 7 cells from KO mice treated with vehicle or MTEP. *P*_drug_ = 0.0328, *P*_genotype_ = 0.0141, and *P*_interaction_ = 0.0417 for sEPSC frequency by two-way ANOVA with Bonferroni’s post hoc test and *P* < 0.0001 by Kolmogorov-Smirnov test. (**B**) Inhibition of mGluR5 by MTEP reduced PPR in KO neurons. *P* < 0.0001 from two-way ANOVA with Turkey post hoc test. *n* = 8 neurons from WT mice treated with vehicle or MTEP and *n* = 7 neurons from KO mice treated with vehicle or MTEP. (**C**) The sEPSC frequency of *Mir501*-KO neurons was reduced by treatment with AP5 or AP5 + MTEP and was similar to that of vehicle- or AP5- and/or MTEP-treated WT neurons. *n* = 8 cells from WT mice and *n* = 7 cells from KO mice. *P*_drug_ = 0.0010, *P*_genotype_ = 0.0006, and *P*_interaction_ = 0.0001 for sEPSC frequency by two-way ANOVA with Bonferroni’s post hoc test and *P* < 0.0001 by Kolmogorov-Smirnov test. All data represent the means ± SEMs. *P < 0.05, ***P < 0.001, and ****P < 0.0001.

Because substantially increased mGluR5 protein levels were observed in *Mir501*-KO mice, we then examined whether inhibition of mGluR5 activity would diminish the behavioral and synaptic deficits associated with the loss of miR-501. Thus, we used MTEP, a negative allosteric modulator of mGluR5 (*21*), to block the activity of mGluR5 in mice. We observed that acute administration of MTEP (3 mg/kg of body weight) improved impaired social preference (Fig. 9, A and B), social novelty recognition (Fig. 9, C and D), and novel object recognition (Fig. 9E) in *Mir501-*KO mice but displayed no effect on their WT littermate mice. Moreover, *Mir501*-KO mice chronically administrated with suboptimal dose of MTEP (3 mg/kg of body weight for 5 day) displayed similar acoustic startle response as WT littermates, both of which displayed higher PPI at 78, 78, 82, 86, and 90 dB than KO mice administrated with vehicle (Fig. 9F). Next, we tested whether the above pharmacological diminishment of abnormal behaviors in *Mir501*-KO mice involved normalization of enhanced excitatory synaptic transmission. We observed that MTEP treatment of *Mir501*-KO hippocampal neurons normalized the increased sEPSC frequency (Fig. 10A) and PPR (Fig. 10B) to levels comparable to those of WT neurons. In contrast, MTEP had no such effects on WT neurons.

Because mGluR5 acts synergistically with NMDAR (*21*), we next determined whether NMDARs are involved in mGluR5-associated increases in excitatory synaptic transmission. Bath application of the specific NMDAR antagonist 2-amino-5-phosphonopentanoic acid (AP5; 50 μM) in hippocampal pyramidal neurons normalized the increased sEPSC frequency observed in *Mir501*-KO mice (Fig. 10C, top right). In these neurons, subsequent application of MTEP failed to alter the frequency of sEPSCs in the presence of AP5 (Fig. 10C). In contrast, we did not observe such effects on WT neurons. Together, these results suggest that abnormal sociability and memory behaviors in *Mir501-*KO mice can be attenuated by normalizing the excitatory synaptic transmission of hippocampal pyramidal neurons through inhibition of the mGluR5/NMDARs activity.

## DISCUSSION

Schizophrenia is a complex polygenetic disease and is characterized by extreme heterogeneity. miRNAs perform their regulatory function by binding to the 3′UTRs of many targets, and as altered miRNA profiles have been implicated in schizophrenia (*7*–*18*), the functional contribution of molecular networks and interactions among many targets of individual miRNAs to the pathogenesis of schizophrenia is largely unknown. In this study, we used SDC MZ twins to screen DE-miRNAs associated with disease susceptibility of MZ twins. As phenotype-discordant MZ twins share a common genetic background, analysis of MZ twin to control for similar genetic background adds to the strength of this study in phenotype variability through changes in epigenetic profiles. We then used multiple rigorous approaches to translating findings in patients with schizophrenia to an animal model of disease and delineated the epigenetic mechanism by which the loss of schizophrenia-associated down-regulated miR-501-3p in mice induced sociability, memory, and sensorimotor gating disruptions through mGluR5-mediated excitatory glutamatergic transmission enhancement, providing etiological implications for schizophrenia.

In this study, transcriptomic analysis of peripheral blood from three pairs of SDC twins detected 15 schizophrenia-associated DE-miRNAs, and 10 miRNAs remained consistently altered in schizophrenia after four HCC twin pairs were included to control for the nonpsychiatric-related trait differences between MZ twins, which was also used in our recent study (*2*). Notably, the findings of two independent peripheral blood studies using sRNA-seq or miRNA microarray (*9*, *12*) showed that 5 of 10 schizophrenia-associated miRNAs were consistently down-regulated (FDR < 0.1) in schizophrenia, supporting that a study of epigenetic profiles in a small sample size of MZ twins could successfully identify epigenetic changes associated with complex traits (*2*). In general, analysis of gene expression is typically better powered to detect increased abundance rather than decreased abundance; however, it is unexpected that 9 of 10 schizophrenia-associated DE-miRNAs identified in MZ twins displayed reductions in schizophrenia, indicative of other epigenetic mechanisms underlying the regulation of miRNA expression in MZ twins. This notion is supported by a previous study showing that a cluster of schizophrenia-associated miRNAs transcribed from imprinted *DLK-DIO3* region of 14q32 displayed significant reductions in PBMCs of schizophrenia (*9*). Our recent study also observed that allele-specific DNA methylation at regulatory loci, including a small number of known imprinted loci, displayed schizophrenia-associated switching in phenotype-discordant MZ twins (*30*), further suggesting a vestige of broader epigenetic alterations to posttranscriptional regulation. Of 10 schizophrenia-associated DE-miRNAs identified in MZ twins, miR-501-3p displayed consistent schizophrenia-associated reduction in both PBMCs (FDR = 0.0003) and postmortem brain tissues (FDR = 0.025) in previous two independent studies (*12*, *13*). This reduction was further validated by qRT-PCR in PBMCs of another independent cohort in our study. In addition, miR-501-3p is highly expressed in multiple tissues and specific neuronal types (*17*, *40*–*42*) and in the mouse cortex and hippocampal brain region examined in our study and in previous publications (*38*, *39*), indicating that miR-501-3p is a brain-relevant miRNA. miR-501-3p displayed consistent schizophrenia-associated down-regulation in both brain and peripheral blood, suggesting that miR-501-3p may be a potential biomarker. This finding is supported by the logistic regression analysis showing that blood miR-501-3p expression levels could distinguish people with schizophrenia from nonpsychiatric controls in an independent Chinese validation cohort. We also observed that two subthreshold GWAS single-nucleotide polymorphisms [rs13059327 (*P* = 1.18 × 10^−3^) and rs181726666 (*P* = 8.75 × 10^−3^)] located upstream of *MIR501* (*37*) and two ultrarare protein-altering variants in its host *CLCN5* gene (ENST0000376088; c.1049G>A and c.2248C>T) (*50*) were associated with schizophrenia. Previously, the serum miR-501-3p level has also been observed to be down-regulated in patients with AD and observed to be correlated with disease progression (*29*), and the expression level of this miRNA in the peripheral circulation is also considered one of the promising miRNAs to predict cognitive performance (*51*). Considering that AD and schizophrenia share many common symptoms, such as cognitive defects, social withdrawal, and delusions (*52*–*54*), more clinical data testing the association specificity of miR-501-3p with cognitive function or either disease is needed. In addition, as circulating Ago2 complexes serve as a significant carrier of miRNA in plasma (*55*), extracellular sources may also provide additional insight into the use of miR-501-3p expression in peripheral tissues as a biomarker.

We delineated the epigenetic and pathophysiological mechanisms by which loss of miR-501 in mice resulted in synaptic and behavioral deficits through mGluR5-mediated excitatory glutamatergic transmission enhancement. Emerging evidence suggests that dysfunction of glutamatergic receptors is key to the etiology of schizophrenia (*21*). Glutamate is an excitatory neurotransmitter present at most synapses in the brain, and it is vital to neurodevelopment as well as synapse organization and sensorimotor gating in adults. Changes in dendritic structure and synaptic organization are associated with thousands of proteins and are ultimately responsible for learning, memory, cognition, and other brain functions (*24*), which are also associated with schizophrenia (*23*, *24*). Thus, it is critical to identify the gene networks that modulate dendritic structure, synaptic transmission, and neuron development. Given that many miRNAs target glutamate synaptic transmission (*6*, *7*, *18*–*20*), the implication of synaptic plasticity in cognition raises the possibility that miRNAs contribute to the pathogenesis of schizophrenia through regulation of synaptic plasticity. However, this possibility needs to be consolidated by more experiments testing functions of risk miRNAs. In this study, we observed that loss of miR-501-3p in mice induced impairments in dendritic structure and growth, sociability, memory and sensorimotor gating, and excitatory glutamatergic transmission. These impairments were ameliorated when miR-501-3p expression was rescued in brain neurons. Previously, miR-501-3p was also identified as a regulator of AMPAR subunit GluA1 expression (*20*). miR-501-3p expression is up-regulated locally in dendrites following NMDAR subunit GluN2A activation, and this regulation is responsible for maintaining the NMDA-dependent suppression of GluA1 expression and long-lasting remodeling of rat dendritic spines (*20*). However, we did not observe GluA1 alterations in the transcriptomic or proteomic analysis of *Mir501*-KO mouse brains, while GluN2A and GluN2B were observed to be increased in the immunoblot analysis of synaptosome fractions of *Mir501*-KO mouse brains, indicating that miR-501-3p may also target other synaptic proteins or pathways. These results were further supported by the proteomic results showing that several well-known schizophrenia risk genes encoding the proteins mGluR5, Gabrb2, and Shank3, which are involved in modulating chemical synaptic transmission, transsynaptic signaling, and synaptic signaling pathways, were identified as targets of miR-501-3p and were altered in *Mir501*-KO mouse brains. However, most of the targets up-regulated in *Mir501*-KO mice at the protein level showed no significant changes at the RNA level in our *Mir501*-KO mice and in previously reported miR-501-3p–overexpressing SK-N-SH cell lines (*29*), shedding light on important regulatory roles of miR-501-3p in posttranscriptional gene expression associated with synaptic dysfunction underlying schizophrenia.

Moreover, mGluR5, a type 1 metabotropic glutamate receptor 5, was identified as one of the direct targets of miR-501-3p. As a candidate schizophrenia risk gene (*21*), mGluR5 has also been reported to be up-regulated in the postmortem brain tissue of patients with schizophrenia at the protein (*27*) and RNA levels (*28*). Inhibition of mGluR5 activity with MTEP in *Mir501*-KO mice ameliorated sociability, memory, and sensorimotor gating deficits and normalized enhanced excitatory synaptic transmission induced by the loss of miR-501-3p. Previously, mGluR5 was reported to be associated with cognitive and social behaviors and conditioning of the sensorimotor gating process by enhancing NMDAR function (*45*–*49*). MTEP was also reported to normalize the group I mGluR-induced spontaneous glutamate release at a central auditory synapse (*56*). This is reminiscent of another mGluR5-negative allosteric modulator, 2-methyl-6-(phenyl ethynyl) pyridine (MPEP), which normalizes social interaction and NMDA/AMPA imbalance in *IRSp53*^−/−^ mice (*47*). In addition, miR-501-3p expression decreases with the postnatal development of the rat hippocampus (*20*), whereas mGluR5 expression increases with postnatal development (*57*), indicating that miR-501-3p is involved in developmental regulation of mGluR5 expression. Moreover, activation of mGluR5 alone does not induce spine shrinkage, which requires the simultaneous activation of metabotropic signaling by NMDARs through the mechanistic target of rapamycin complex 1 signaling pathway (*58*). Our study also found increased protein levels of mechanistic target of rapamycin from the TMT assay and GluN2A and GluN2B from the immunoblot assay in *Mir501*-KO mouse brains. These reports, together with observations that the NMDAR antagonist AP5 also normalized the increased sEPSC frequency in *Mir501*-KO mice, suggest that, in addition to mGluR5/NMDAR, multiple synaptic signaling pathways contribute to behavioral deficits in *Mir501*-KO mice. However, although we did not observe the effects of MTEP or AP5 on WT mouse phenotypes, it is still possible that modulating mGluR pathways by MTEP or AP5 independently affected these functional readouts from any effects by the loss of miR-501-3p.

Several direct evidence implicating miRNAs in neurological processes are derived from KO studies in rodents (*18*, *59*); however, the consequences of “marked” genetic modulations in the context of a mouse genetic background do not completely replicate the human condition. Therefore, a conditional model may be a better choice in future studies. Although schizophrenia-associated miR-501-3p down-regulation was identified from peripheral blood in our study, multiple lines of evidence reveal that miR-501-3p is a brain-relevant miRNA, and brain-derived exosomal miR-501 also showed a significant reduction in patients with schizophrenia, providing a functional relevance of dysregulated miR-501-3p expression to the pathophysiology of schizophrenia. Moreover, conditional rescue of *miR501* expression in central nervous system neurons ameliorating behavioral and synaptic deficits induced by loss of miR-501 further established the links of miR-501-3p to the modulation of glutamatergic transmission in a rodent mouse model; however, further studies in specific neuronal or circuity function confirm that the role of miR-501-3p in the pathophysiology of schizophrenia is needed. In addition, it will also be interesting to determine whether *Mir501-*KO female mice could display behavioral and synaptic deficits observed in *Mir501-*KO male mice although the *Mir501* gene is located on chromosome X and circulating ovarian hormones might make data from female animals more complex and variable than data from males (*60*). Last, it would be interesting to validate the functions of other DE-miRNAs identified in this study.

In summary, our findings suggest an epigenetic mechanism by which suggests that the down-regulation of miR-501-3p may lead to molecular, cellular, electrophysiological, and behavioral abnormalities associated with schizophrenia through mGluR5-mediated excitatory glutamatergic transmission enhancement. This epigenetic and pathophysiological mechanism that links miR-501-3p to the modulation of excitatory glutamatergic transmission has etiological implications for schizophrenia.

## MATERIALS AND METHODS

### Human participants

Peripheral blood samples from two independent Han Chinese cohorts, including a discovery cohort and validation cohort, were used in this study (table S1). The discovery cohort included three pairs of SDC twins and four pairs of HCC twins, whose blood RNA samples were examined by sRNA-seq. The validation cohort included 136 patients with schizophrenia and 76 unrelated nonpsychiatric controls whose blood RNA samples were examined by qRT-PCR. All participants fulfilled the diagnostic criteria for schizophrenia according to the *Diagnostic and Statistical Manual of Mental Disorders* (American Psychiatric Association, ed. 4). The nonpsychiatric controls were free from any present, past, or family history of mental illness or substance abuse diagnosis. Approval for the work was obtained from the local medical ethics committees of the participating hospitals. All participants signed informed consent before the study following the presentation of the nature of the procedures.

### Animals

All mice used were from the C57BL/6J genetic background. The *Mir501^fx/fx^* (KO) targeting vector was designed by inverting the *Mir501* gene and flanking it with the FLEx cassette (*61*), which is composed of one pair of loxP sites staggered with one pair of lox2722 sites (Fig. 2A). *Mir501^fx/fx^* (female) or *Mir501^fx/y^* (male) conditional knock-in mice were generated by homologous recombination in R1 embryonic stem cells and implanting of the correctly targeted cells into C57 blastocysts using standard procedures in Shanghai Model Organisms Center (Shanghai, China). Male chimeras were crossed with C57BL/6J females to generate heterozygotes (*Mir501^fx/y^* for males or *Mir501^fx/+^* for females) for at least two generations before the generation of experimental animals. To generate experimental animals, all germline *Mir501^fx/y^* (KO) male mice along with their respective WT (*Mir501^+/y^*) littermates were produced by breeding *Mir501^fx/+^* mice with *Mir501^fx/y^* mice (Fig. 2C). For the *Mir501*-RE experiments (Fig. 5A), *Mir501^fx/+^:Cre^−/−^* mice were crossed with *Mir501^+/y^:Nestin-Cre^+/−^* [B6.Cg-Tg(Nes-cre)1Kln/J] mice (003771, the Jackson Laboratory) to generate RE (*Mir501^fx/y^:Nestin-Cre^+/−^*) mice along with their male littermate KO (*Mir501^fx/y^:Nestin-Cre^−/−^*) and WT (*Mir501^+/y^:Nestin-Cre^+/−^* and *Mir501^+/y^:Nestin-Cre^−/−^*) mice. DNA extracted from mouse tails was used for genotyping using the specific primers WT forward (WT-F) and WT reverse (WT-R) or KO-F and KO-R (table S8). The environmental conditions were kept constant: food and water ad libitum, 21° ± 0.5°C, 60 ± 10% relative humidity, 12-hour light/dark cycles, and three to five mice per cage as previously described (*2*). Considering that the *Mir501* is located on the X chromosome, this study used male animals in all experiments. The mice were euthanized by cervical dislocation after anesthesia under a halothane atmosphere. All animal protocols were approved by the Animal Care and Use Committees of Southern Medical University. Mice were collected at P90 for histological analysis. Major organ tissues, including the kidneys, brain, liver and heart, were dissected immediately after the animals were euthanized and fixed in 4% paraformaldehyde at 4°C overnight, stored in 70% ethanol, and embedded in paraffin. The paraffin blocks were sectioned at 5 μm. After deparaffinization, the sections were stained with hematoxylin and eosin or Nissl stain using the standard procedure.

### RNA or sRNA-seq analysis

TRIzol reagent (Invitrogen, Carlsbad, CA, USA) was used for RNA extraction from PBMCs. RNA integrity was assessed with the Bioanalyzer 2100 RNA 6000 Nano Kit (Agilent Technologies, Santa Clara, CA, USA). All RNA with a minimum RNA integrity value of seven was used for library preparation (*2*). The library was sequenced to a depth of ~12 million 50-bp single-end reads per sample on an Illumina HiSeq 2000 for sRNA-seq by BGI Solutions (Shenzhen, China). After quality control using FastQC, the reads from sRNA-seq were aligned to the University of California, Santa Cruz *Homo sapiens* reference genome hg19 using Bowtie (*62*). Each miRNA from miRBase Sequence Database 21 (*31*) was counted using miRDeep2 (*32*). We used Cuffdiff to convert the counts into total TPM mapped read values, which represented the miRNA expression levels. Low-abundance miRNAs that were not measurable in >50% (TPM < 0) of samples were excluded from further analysis. To screen the DE miRNAs between SDC twins, we used edgeR (|logFC| > 0.585 and *P* < 0.05) and further performed case-control analysis of three schizophrenia cases (from three SDC twin pairs) versus 11 healthy controls by including four HCC twin pairs (|logFC| > 0.585 and *P* < 0.05) based on the strategy in our recent study (*2*).

RNA extracted from P90 mouse cortical tissues using TRIzol reagent (Invitrogen) with a minimum RNA integrity value of 7 was used to build an RNA library as previously described (*2*). Briefly, the library was sequenced to a depth of ~27 million 150-bp paired-end reads per sample on an Illumina HiSeq X Ten by Novogene Solution. The raw reads were subjected to quality control with FastQC, and clean reads generated from the raw reads with Trimmomatic were mapped to the GRCm38 mouse assembly in the Ensembl database using TopHat2. We used Cufflinks to quantify the expression levels as fragments per kilobase of exon model per million mapped fragments (FPKM) as values and then used Cuffdiff with FDR correction and an FC method to identify DEGs between WT and KO mice.

### Quantitative reverse transcription polymerase chain reaction

The total RNA extracted from hippocampal tissue or PBMCs using TRIzol reagent (Invitrogen, USA) was reverse-transcribed into complementary DNA (cDNA) using a Bulge-Loop miRNA RT Primer for subsequent miRNA quantification and oligo(dT) primers for mRNA quantification with the PrimeScript RT Reagent Kit with gDNA Eraser (TaKaRa, Japan). A comparative qPCR assay was performed with a SYBR Premix Ex Taq II kit (TaKaRa) on a LightCycler 96 System (Roche) with *U6* (for miRNA) or *Gapdh* (for mRNA) as a reference gene for the quantification of target miRNA or mRNA levels, and the estimated values expressed as 2^−ΔΔ*C*t^ values were subjected to statistical analyses.

### In situ hybridization

In situ hybridizations were performed by adapting miRNA localization protocols (Exonbio, Guangzhou, China). Brain tissue was rinsed in phosphate-buffered saline (PBS) and fixed with 4% formaldehyde (w/v) in PBS overnight at 4°C. After fixation, the samples were dehydrated in 100% ethanol overnight and were embedded in paraffin. Sagittal sections of the whole brain were prepared at 4 μm, collected onto slides, and air-dried overnight at room temperature. The mmu-miR-501-3p fluorescence in situ hybridization (FISH) probe (Exonbio) was double-labeled with 5′-digoxigenin (DIG) and 3′-DIG and hybridized to brain sections at 55°C, and the sections were stained with an anti-digoxin horseradish peroxidase (HRP)–conjugated secondary antibody and fluorophore-conjugated tyramine using a miRNA FISH kit (Exonbio). For all of the miR-501 in situ experiments, at least three independent experiments were conducted. Representative images are shown in the figure.

### Golgi staining

Golgi staining was performed as previously described using a kit from FD NeuroTechnologies. Briefly, brains from four pairs of independent littermate *Mir501*-KO, *Mir501*-WT, or *Mir501*-RE mice were harvested whole and rinsed with distilled water. The brains were incubated in a 1:1 mixture of FD solution A: solution B for 24 hours at room temperature in the dark. The brains were then transferred to fresh FD solution A:B for 2 weeks at room temperature in the dark. The brains were then transferred into FD solution C and stored in the dark at room temperature for 48 hours. Coronal sections (150 μm) were cut with a Leica CM1950 cryostat and mounted on 3% gelatin-coated slides. Staining was performed according to the manufacturer’s protocol, and slides were dehydrated in ethanol and mounted with Premount medium. The dendrites were traced, and their lengths were measured using the Fiji plugin NuronJ. The spines were counted individually on secondary branch dendrites in the DG hippocampal region. The spine densities were quantified by tracing the dendritic segments, in which the spines were counted. The dendritic spines were categorized into five types (stubby, thin/filopodial, mushroom-shaped, branched, and multiple branched) as previously described (*63*).

### Image analysis

Z-stack images (at 1-μm intervals for Sholl analysis or 0.3-μm intervals for spine analysis) were obtained on a BX63 microscope (Olympus, Japan) with a 40× or 100× oil immersion lens. Image analyses and quantification were performed using Fiji software. For photomicrographic illustration of dendritic spines, the images were processed with cellSens Dimension software (Olympus) to deblur the images caused by spines oriented in different depths of focal planes and enhanced with Photoshop CC 2018 software (Adobe).

### Behavioral procedures

Mice were housed in groups of three to five animals in our institutional animal facility with a 12-hour light cycle (lights on at 8:00 a.m.) with ad libitum access to food and water, and all behavioral tests were performed from 10:00 a.m. to 17:00 p.m. as previously described (*2*). Briefly, on the test day, the mice were transferred to the testing room and acclimated to the room conditions for at least 1 hour. After each individual test session, the apparatus was thoroughly cleaned with 70% alcohol to eliminate the odor and trace of the previous tested mouse. All behavioral procedures were performed with 3-month-old male mice obtained from at least two independent cohorts. Animal experiments were in full compliance with the National Institutes of Health Guide for Care and Use of Laboratory Animals and were approved by the Institutional Animal Care and Use Committee at Southern Medical University. MTEP (HY-13206, MedChemExpress) dissolved in 10% Tween 80 (v/v) and 90% sterile saline. Intraperitoneal injection of MTEP (3 mg/kg) was performed 10 min before the behavioral test. Previous study demonstrated that acute systemic administration of MTEP (3 mg/kg, intraperitoneal, mice) could maintain >50% mGluR5 receptor occupancy for 30 min and lead to significant receptor blockade in the brain (*64*). For PPI test, MTEP was administrated intraperitoneal injection of 3 mg/kg for five continuous days, and the last injection was performed 10 min before the PPI test.

#### *Three-chamber social test*

Testing was conducted in a rectangular three-chambered box with small square openings (5 cm by 5 cm) between the chambers (each compartment was 20 cm by 40 cm by 22 cm). The outside walls of the chamber were opaque, while the inner dividers were clear plexiglass. After a 5-min habituation period in the empty chamber, the test mouse was moved into the empty center chamber with the partitions in place. A wire mesh cup containing a novel mouse (S1) was placed on one side of the chamber, and an empty mesh cup (E) was placed on the other side during the “sociability” session. The partitions were then lifted, and the test mouse was free to explore all the sections of the chamber. After 10 min, the test mouse was moved into the empty center with the partitions in place. A second novel mouse (S2) was placed under the empty cup, the partitions were removed, and the test mouse was allowed to freely explore the chamber during the “social novelty preference” session for 10 min. The time spent in each of the three chambers during each test session was quantified using LabMaze V3.0.

#### *Novel object recognition test*

Mice were individually placed in a Plexiglas arena (50 cm by 50 cm by 40 cm) for 5 min, and exploration was quantified by video tracking as previously described (*2*). Subsequently, the mice were subjected to habituation sessions in which two objects identical in shape, color, and odor were introduced into the arena for 10 min. The mouse was placed in the holding cage before the next session. One hour later, one of the objects (O1) was replaced with a novel object (O2). The time spent in active exploration of each object was scored during each session from video recordings (LabMaze V3.0).

#### *PPI test*

PPI tests were performed as previously described (*2*). The experiments were performed with a sound-attenuating test chamber (65 cm by 35 cm by 25 cm). Mice were placed in a plexiglass tube mounted on a plastic frame, and motion was monitored using a piezoelectric accelerometer. Each chamber was equipped within a commercial startle reflex system (SR-LAB, San Diego Instrument, CA, USA). Each test session began with a 5-min acclimation period in the presence of 70-dB acoustic background noise. During the test, mice were subjected to six startle trials (40 ms of 120 dB of white noise) and six prepulse/startle trials (20 ms of white noise at 74, 78, 82, 86, or 90 dB; a 100-ms interval; and 40 ms of white noise). Different trial types were presented pseudo-randomly, with each trial type presented six times, and no two consecutive trials were identical. Mouse movement was measured for 100 ms after startle stimulus onset (sampling frequency, 1 kHz) for 100 ms. The PPI (%) was calculated with the formula 100 × (startle amplitude for pulse alone-startle amplitude for pulse with prepulse)/startle amplitude for pulse alone.

### Electrophysiological recording

Three-month-old mice were anesthetized with pentobarbital sodium (50 mg/kg) and then quickly decapitated to remove the brains into ice-cold cutting solution (containing 2 mM KCl, 1.25 mM NaHPO_4_, 0.2 mM CaCl_2_, 12 mM MgSO_4_, 10 mM d-glucose, 220 mM sucrose, and 26 mM NaHCO_3_; oxygenated with 95% O_2_ + 5% CO_2_). Coronal brain slices containing the hippocampus (300 μm) were prepared with a vibratome (VT1200S, Leica). After sectioning, the slices were incubated in standard artificial cerebrospinal fluid (ACSF) (containing 126 mM NaCl, 2.5 mM KCl, 1.25 mM NaHPO_4_, 2 mM CaCl_2_, 10 mM d-glucose, and 26 mM NaHCO_3_; oxygenated with 95% O_2_ + 5% CO_2_) for 20 to 30 min at 34°C and then recovered for at least 1 hour at room temperature before recording. For drug treatments in the electrophysiological recording experiments, all drugs were freshly prepared in ACSF before the experiments and delivered via pumps to reach their final concentrations. MTEP and AP5 were used in the sEPSC recording experiments at concentrations of 20 and 50 μM during recording.

### sEPSC, sIPSC, and PPR analyses

Whole-cell patch-clamp recordings were performed using a MultiClamp 700B amplifier, with signals being recorded/analyzed using a Digidata 1440A data acquisition system and the pClamp 10.7 software package (Molecular Devices). Recordings were conducted on visually identified pyramidal neurons of the CA1 region using patch pipettes that had a resistance of 4 to 6 megohms when filled with intracellular solutions. sEPSCs and evoked EPSCs were voltage-recorded with pipette solution (containing 135 mM K-gluconate, 5 mM KCl, 10 mM Hepes, 2 MgCl_2_, 0.2 mM Na_2_-ATP, and 2 mM MgCl_2_) at a holding potential of −70 mV in ACSF containing 10 μM SR95531 to block γ-aminobutyric acid type A receptors. sIPSCs and evoked IPSCs were voltage-clamped with a pipette solution [containing 110 mM Cs_2_SO_4_, 0.5 mM CaCl_2_, 2 mM MgCl_2_, 5 mM EGTA, 5 mM Hepes, 5 mM triethylammonium (TEA), and 5 mM Mg-ATP] at 0 mV in ACSF containing 50 μM AP5 and 20 μM 2,3-dihydroxy-6-nitro-7-sulfamoylbenzo[*f*]quinoxaline to block glutamate receptors. Paired postsynaptic currents were elicited with pairs of identical stimuli separated by intervals of 25, 50, 100, 150, and 200 ms with a bipolar nichrome-stimulating electrode positioned at the Schaffer collaterals.

### Intrinsic excitability

To characterize neuronal intrinsic excitability, cells were current-clamped with the same pipette solution used in recording the sEPSCs. The RMP was determined at *I* = 0 mV immediately after breakthrough of cells. The membrane input resistance was calculated using Ohm’s law through delivery of a −10-pA hyperpolarizing current with a 500-ms duration to each cell. The rheobase was defined as the minimum current that elicited the first AP. The AP threshold was defined as the onset potential of the rapidly rising phase of the spike.

### Quantitative analysis of the proteome by multiplexed TMT-two-dimensional liquid chromatography-tandem mass spectrometry

Quantitative proteomics analysis by a TMT strategy was conducted by Novogene. Briefly, P90 mouse cortical tissues were dissected and immediately homogenized in buffer [8 M urea and 100 mM NH_4_HCO_3_ (pH 8.0)] in a matrix D tube on a Fast-Prep 24 homogenizer. The lysate was centrifuged at 12,000*g* for 15 min at 4°C, and the supernatant was reduced with 10 mM dithiothreitol for 1 hour at 56°C and subsequently alkylated with sufficient iodoacetamide for 1 hour at room temperature in the dark. The samples were precipitated by adding 4 volumes of cold acetone (−20°C) and incubated at −20°C overnight. The precipitated samples were centrifuged at 12,000*g* for 15 min at 4°C. After washing with 1 ml of cold acetone, the pellet was dissolved in dissolution buffer [8 M urea and 100 mM TEA bicarbonate (TEAB) (pH 8.5)]. After quantification by Bradford protein assay and protein integrity evaluation by SDS–polyacrylamide gel electrophoresis, 200 μg of protein aliquots from each sample were adjusted to 2 μg/μl with dissolution buffer [8 M urea and 100 mM TEAB (pH 8.5)] and digested at 37°C for 4 hour. After that, the samples were diluted with trypsin and CaCl_2_ for an additional overnight incubation before being quenched with 1% trifluoroacetic acid. The following peptide desalting, TMT labeling, and efficiency testing procedures were performed according to the manufacturers’ procedures.

Equally mixed TMT-labeled peptides from all samples were desalted and then fractionated in a C18 column (Waters BEH C18; 4.6 mm by 250 mm, 5 μm) on a Rigol L3000 high-performance liquid chromatography (HPLC) system using a gradient elution developed with mobile phases A [2% acetonitrile (pH 10.0)] and B (98% acetonitrile). The column temperature was set at 45°C. Approximately 10 fractions were finally collected. Each fractionated peptide was dried under vacuum and reconstituted in 0.1% (v/v) formic acid in water, after which the peptides were run individually and sequentially without concatenation. The samples were analyzed on an EASY-nLC 1200 UHPLC system (Thermo Fisher Scientific) coupled with a Q Exactive series mass spectrometer (Thermo Fisher Scientific) operating in the data-dependent acquisition mode. A 1-μg sample was injected into a C18 Nano-Trap column (4.5 cm by 75 cm, 3 μm). The peptides were separated in an analytical column using a linear gradient. The separated peptides were analyzed with a Q Exactive series mass spectrometer (Thermo Fisher Scientific) with a Nanospray Flex ion source (ESI). The full scan range was from a mass/charge ratio (*m*/*z*) 350 to 1500 with a resolution of 6000 (at *m*/*z* of 200), an automatic gain control (AGC) target value of 3 × 10^6^, and a maximum ion injection time of 20 ms. The top 40 precursors of the highest abundance in the full scan were selected, fragmented by higher-energy collisional dissociation, and analyzed via tandem mass spectroscopy (MS/MS). For MS/MS, the resolution was 45,000 (at *m*/*z* of 200) for 10-plex analysis, the AGC target value was 5 × 10^4^, the maximum ion injection time was 86 ms, the normalized collision energy was set to 32%, the intensity threshold was 1.2 × 10^5^, and the dynamic exclusion parameter was 20 s.

The MS data were analyzed using Proteome Discover 2.2 (PD 2.2) and searched against the mouse UniProtKB protein sequence database with an automatically generated decoy database (reverse sequence). The specific parameters included a maximum of two missed cleavages, a 10–parts per million mass tolerance for precursor ions and a 0.02-Da mass tolerance for products. Carbamidomethyl was specified as the fixed modification, oxidation of methionine and TMT plex were specified as the dynamic modifications, and acetylation and TMT plex were specific as the N-terminal modifications in PD 2.2. PD 2.2 software further filtered the retrieval results. Peptide spectrum matches (PSMs) with a credibility of more than 99% were identified as PSMs. The identified proteins contained at least one unique peptide. The identified PSMs and proteins with FDR values of <1.0% were retained and analyzed. The relative protein abundance ratios between groups were calculated using the average of the normalized reporter ion intensities of the group and subjected to a two-tailed Student’s *t* test with the threshold of significance at *P* < 0.05. We identified DEPs with the following criteria: (i) absolute mean *z* score ≥ 1.5, (ii) the same trend in the biological repeat: the signs of *z* score should be consistent, and (iii) *P* < 0.05 from *t* test. The formula of *z* score is *z* score = (expression in KO mouse mean expression in WT mouse)/SD of expression in WT mouse (*18*).

### Integrated bioinformatics analyses

miRNA targets were predicted using miRwalk 2.0 (*65*). miRNA expression and mRNA expression correlations were calculated using the cor function in R (v3.5.1). GO biological process enrichment analysis was performed using ToppGene (*66*). The web-based Gene Set Analysis Toolkit (WebGestalt) was used for additional GO analysis. STRING (https://string-db.org/) was used to predict the functions and interactions of the DEGs. Schizophrenia-associated genes were obtained from Schizophrenia Gene Resource 2 (https://bioinfo.uth.edu/SZGR/) (*35*) and Gene List Automatically Derived For You (GLAD4U) (*36*), as well as CLOZUK and Psychiatric Genomics Consortium (PGC) 2/3 GWAS datasets (*3*, *4*, *37*).

### Dual-luciferase assays

The *Grm5*-WT-3′UTR containing miR-501-3p–predicted targets was PCR-amplified directly from mouse cDNA and then cloned into the 3′ end of the *Renilla* luciferase gene of the psiCHECK-2 dual-luciferase vector containing firefly luciferase driven by a different promoter as an internal control (Promega, Madison, WI, USA) via the Xho I and Not I sites. The binding sites of miR-501-3p in the *Grm5*-3′UTR were predicted using TargetScan and RNAHybrid in miRwalk2.0 (*65*). Mutant reporter constructs (*Grm5*-MT-3′UTR) containing miR-501-3p binding sites GGTGCAT (site 1)– and/or an AGATCC (site 2)–removed 3′UTR were obtained from the *Grm5*-WT-3′UTR reporter using a QuikChange site-directed mutagenesis kit (Stratagene, La Jolla, CA). The miR-501-3p precursor (WT-miR501-3p) was amplified from mouse genomic DNA and cloned into the pEGFPC1 vector via the Hind III and Bam HI sites, and the mutant miR-501-3p (MT-miR501-3p) was generated by removal of the seed region from the WT-miR501-3p expression vector in pEGFPC1. Human embryonic kidney 293T cells were cultured in Dulbecco’s modified Eagle’s medium with 10% fetal bovine serum (Life Technologies) and harvested 48 hours after transfection using Lipofectamine 2000 reagents (Life Technologies), and dual-luciferase activity (Promega) was measured with the Wallac Victor V 1420 Multilabel Counter (PerkinElmer, San Jose, CA, USA) to yield the ratio of *Renilla* luciferase activity to firefly luciferase activity. All luciferase readings were taken from more than three individual biological repeats.

### Western blotting

Synaptosomes were isolated from the whole hippocampus or cortex of the brain for analysis of protein levels. Briefly, the whole hippocampus or cortex was homogenized in 10 volumes of Hepes-buffered sucrose [0.32 M sucrose and 4 mM Hepes (pH 7.4)]. The homogenate was centrifuged at 1200*g* for 10 min to remove cell debris, and the supernatant was centrifuged at 15,000*g* for 20 min at 4°C. The pellets, containing synaptosomes, were gently resuspended in radioimmunoprecipitation assay buffer [50 mM tris (pH 7.4), 150 mM NaCl, 1% Triton X-100, 1% sodium deoxycholate, 0.1% SDS, 2 mM sodium pyrophosphate, 25 mM β-glycerophosphate, 1 mM EDTA, 1 mM Na_3_VO_4_, and leupeptin (0.5 μg/ml)] for further protein analysis. Anti-mGluR5 (AB5675, MilliporeSigma), anti-GluNR2A (19953-1-AP, Proteintech), anti-GluNR2B (21920-1-AP, Proteintech), and anti-PSD95 (20665-1-AP, Proteintech) were used as primary antibodies at a dilution of 1:1000. HRP-labeled secondary antibodies (Proteintech) were used at a dilution of 1:5000. Antibodies against tubulin (10068-1-AP, Proteintech) were used as loading controls. All Western blot quantifications were performed using ImageJ.

### Statistical analysis

Statistical analyses, including two-tailed Student’s *t* test, Mann-Whitney Wilcoxon test (*P*_w_), analysis of variance (ANOVA) with Bonferroni’s post hoc test or Kolmogorov-Smirnov post hoc test, or ANCOVA with age and sex as covariates (*P*_c_), were performed with GraphPad Prism 9.0 or SPSS. The ROC curve in logistic regression models was performed with the R core function to evaluate the discriminant ability of miRNA expression in the sporadic cohort in Fig. 1 (D and E) and with age and sex as covariates in fig. S1E. We also estimated the 10-fold cross-validation (repeat 200 times) accuracy of miRNA expression between groups. Data are presented as the means ± SEM, SD, or 95% confidence interval. The accepted level of significance was *P* < 0.05.

## Notes

View/request a protocol for this paper from *Bio-protocol*.
