# Supplementary material for: Loss of schizophrenia-related miR-501-3p in mice impairs sociability and memory by enhancing mGluR5-mediated glutamatergic transmission
Source: Sci Adv. 2022 Aug 19;8(33):eabn7357. doi: 10.1126/sciadv.abn7357 (PMC9390987; doi:10.1126/sciadv.abn7357)
Supplement: Supplementary file 1 — Figs. S1 to S4 [file sciadv.abn7357_sm.pdf]

Science Advances
AAAS

Supplementary Materials for

Loss of schizophrenia-related miR-501-3p in mice impairs sociability and memory by enhancing mGluR5-mediated glutamatergic transmission

Wenquan Liang *et al.*

Corresponding author: Rongqing Chen, creatego@hotmail.com; Cunyou Zhao, zhaocunyou@gmail.com

Sci. Adv. **8**, eabn7357 (2022)
DOI: 10.1126/sciadv.abn7357

The PDF file includes:

Figs. S1 to S4
Legends for tables S1 to S8

Other Supplementary Material for this manuscript includes the following:

Tables S1 to S8

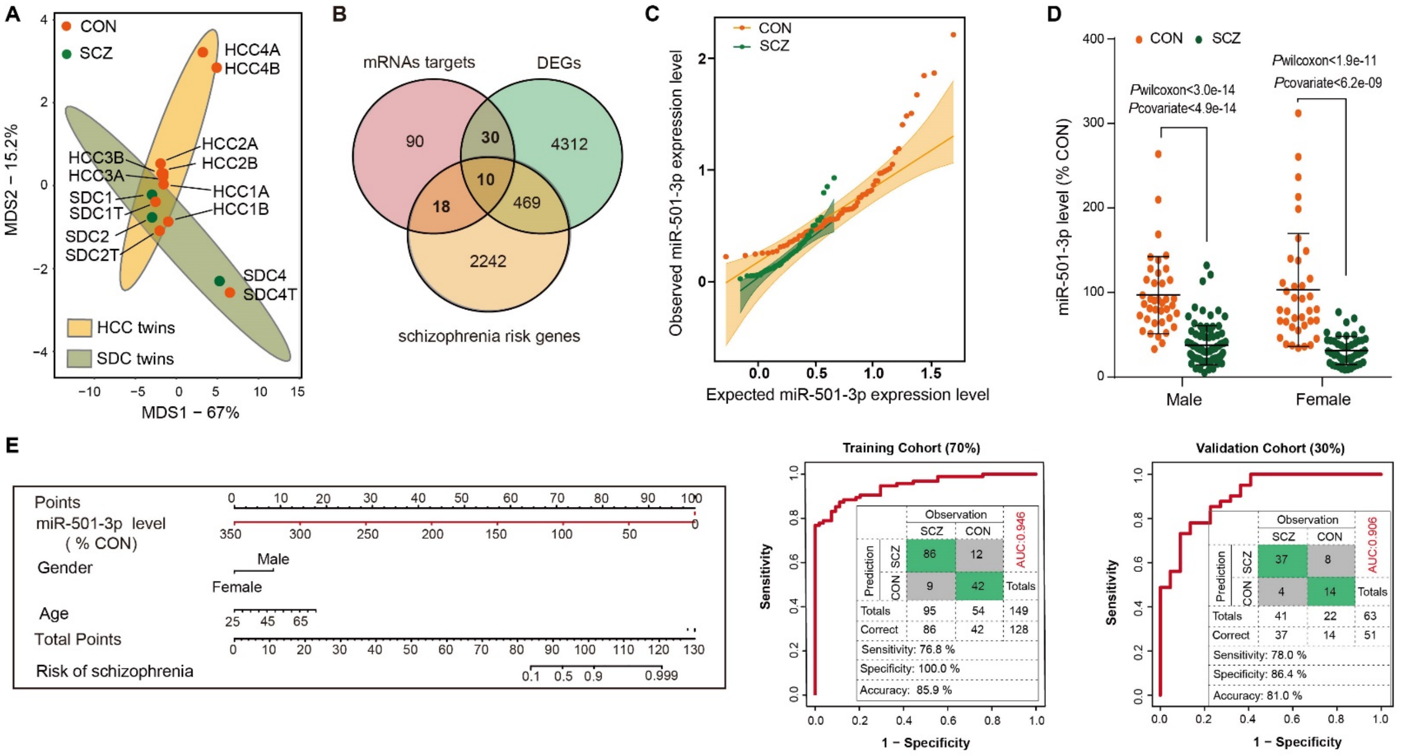

Fig. S1.

miRNAs expression patterns in SCZ and CON. A, MDS plot shows the relationship of 3 SDC with 4 HCC on 240 unique miRNAs expression level using Euclidean distance. **B**, Overlap among 10 DE-miRNAs targets from Ago2-CLIP-seq, schizophrenia risk genes and psychENCODE RNA-seq dataset. **C**, Q-Q plot of normalized miR-501-3p expression level in CON and SCZ groups. **D**, Relative miR-501-3p expression level in separate male and female cohorts of SCZ and CON as shown in **Fig. 1D**. Males: CON (mean=97.1, 95% CI: 75.5-110.9), SCZ (mean=37.9, 95% CI: 28.8-37); Females: CON (mean=103.1, 95% CI: 66.5-107.3), SCZ (mean=31.6, 95% CI: 22.6-38.1). Significant differences between CON and SCZ were determined with Mann-Whitney Wilcoxon test (P_{Wilcoxon}) or with ANCOVA including age as a covariate ($P_{\text{covariate}}$). **E**, Nomogram of binary logistic regression analysis. Three-variable nomogram established by logistic regression for predicting the risk of schizophrenia using transformed miR-501-3p expression level, sex, and age. miR-501-3p expression levels of each individual in panels of D-E are transformed to the mean level in CON.

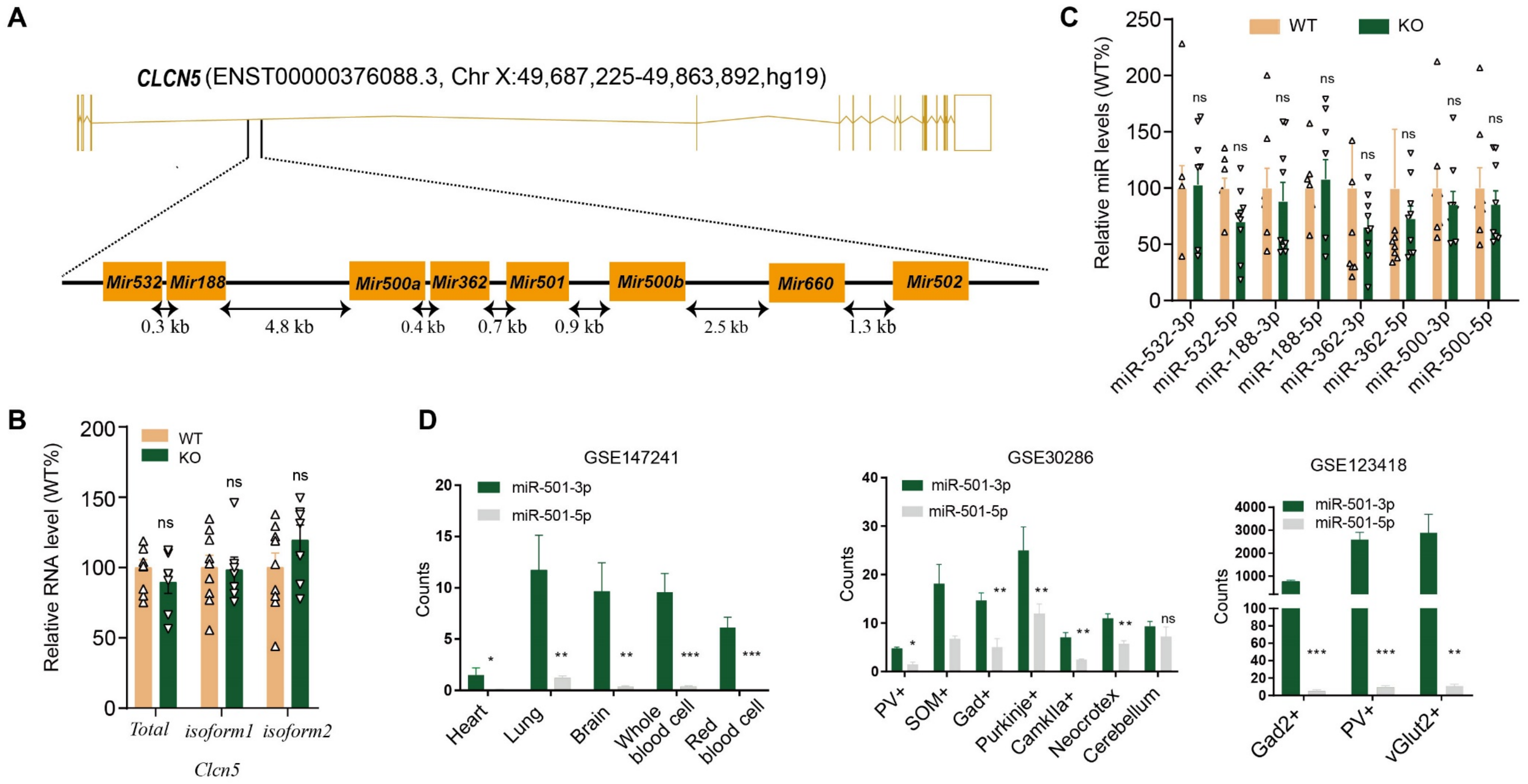

Fig. S2.

***MIR501* gene loci and expression pattern. A**, Schematic representation of human miR-501 locus and its host *CLCN5* gene. **B**,**C,** Expression of *Clcn5* isoforms (**B**) and co-clustered miRNAs (**C**) in WT and KO mouse as measured by qRT-PCR. **D**, miR-501 two mature forms (miR-501-3p and miR-501-5p) expression level in different tissue or neurons. **p*<0.05, ***p*<0.01, ****p*<0.001 or ns, nonsignificant by two-tailed student *t*-test.

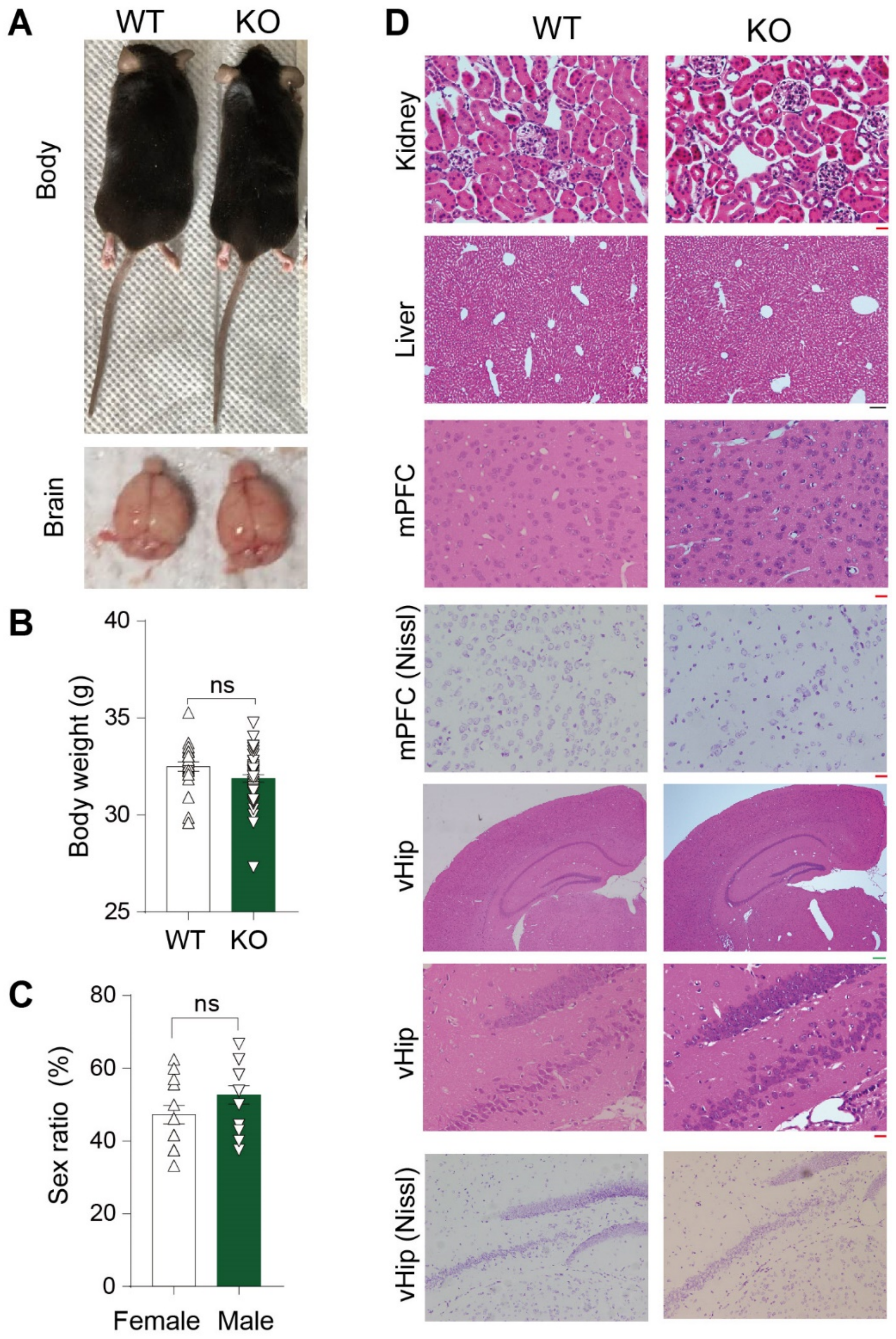

Fig. S3.

Gross appearance and histological sections of miR-501 KO and WT mice. A, Representative WT and KO mice and brain tissues. **B**, The body weight of WT and KO mice at P90 are similar. **C**, The birth sex ratio is similar in WT and KO mice. **D**, No apparent differences in organs' histological sections of kidney and liver tissue (HE staining, 20 x) and slightly change in HE and Nissl staining of mPFC and vHip brain tissues between WT and KO mice.

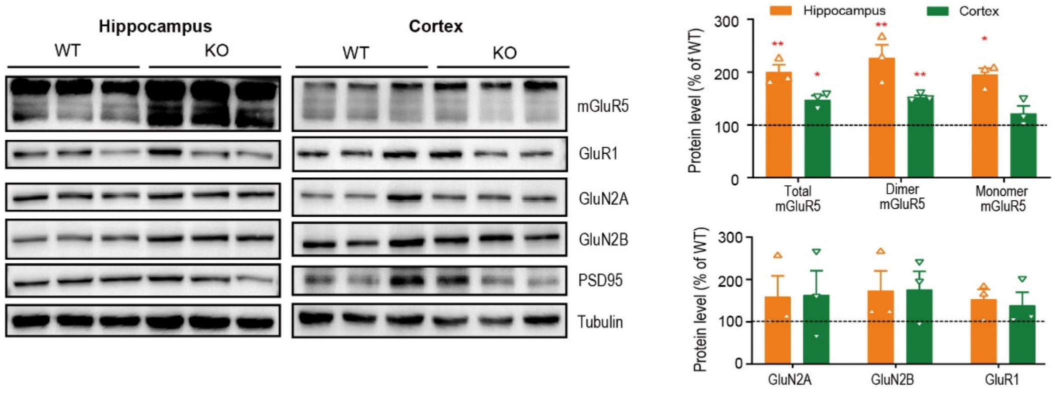

Fig. S4.

Immunoblot assay. Immunoblot analysis of mGluR5, GluNR2A, GluNR2B, PSD95 and Tubulin in the synaptosome fraction of the hippocampus (**left**) and cortex (**middle**) and the relative intensity of each band compared to Tubulin from each of three KOs were normalized to the mean for WT mice (100%, horizontal dashed line; **right**). Significant differences in KO mice compared with WT mice are marked by *p<0.05 or **p<0.01 from two-way ANOVA with Bonferroni's post hoc test above the indicated comparison.

Supplementary Table Legends

Table S1.
Information of subjects employed in this study. Summarized demographic information and sRNA-seq information.

Table S2.
DE-miRNAs identified in MZ twins. Shown with list of 240 unique miRNAs.

Table S3.
Functional enrichment analysis of 148 mRNA targets of 10 DE-miRNAs. Gene Ontology (GO) enrichment analysis was performed using ToppGene shown with GO_BP, GO_CC and Mouse Phenotypes.

Table S4.
DEPs identified from the *Mir501*-KO mice. 165 upregulated DEPs were marked in red fonts, and 60 downregulated DEPs are marked in blue fonts. The seven putative protein targets of miR-501-3p were noted in yellow.

Table S5.
GO biological process analysis of 225 mouse DEPs. GO biological process enrichment analysis was perdormed using ToppGene, and ranked by FDR B&H.

Table S6.
Predicted targets of mmu-miR-501-3p. Predicted targets of mmu-miR-501-3p were obtained from miRwalk 2.0.

Table S7.
DEGs identified from the *Mir501*-KO mice. 452 DEGs ($p<0.05$) were marked in red.

Table S8.
Primers and probes employed in this study. WT-F, WT-R, KO-F, and KO-R were used for genotyping of mouse. Cre-F and Cre-R were used for identification of Cre. pEGFP-C1-mmu-Mir501-F and pEGFP-C1-mmu-Mir501-R were used to construct the overexpression vector of miR-501-3p. psiCHEK2-Grm5-3'UTR-F and psiCHEK2-Grm5-3'UTR-R were used to construct the reported vector of Grm5. Gapdh-F and Gapdh-R were used as reference gene primer in the qRT-PCR.

These tables are attached externally.
